# Clinical Benefits and Safety of Multiple Micronutrient Supplementation During Preconception, Pregnancy, and Lactation: A Review

**DOI:** 10.1093/nutrit/nuaf079

**Published:** 2025-07-14

**Authors:** Jue Liu, Konstantinos Mantantzis, Ligaya Kaufmann, Zigor Campos Goenaga, Olga Gromova, Keiji Kuroda, Hongbo Qi, Nana Tetruashvili, Gian Carlo Di Renzo

**Affiliations:** Department of Regulatory, Medical, Safety, Quality & Compliance (RMSQC), Bayer Healthcare Company Limited, 200126 Shanghai, China; Department of Regulatory, Medical, Safety, Quality & Compliance (RMSQC), Bayer Consumer Care AG, 4052 Basel, Switzerland; Department of Regulatory, Medical, Safety, Quality & Compliance (RMSQC), Bayer Consumer Care AG, 4052 Basel, Switzerland; UMAE HGO4 Luis Castelazo Ayala, Tizapan San Ángel, 01090 Mexico City, Mexico; FRCCSC RAS - Federal Research Center Computer Sciences and Control Russian Academy of Sciences, 119333 Moscow, Russian Federation; Sugiyama Clinic Marunouchi, Center for Reproductive Medicine and Endoscopy, Tokyo, 100-0005, Japan; Department of Obstetrics and Gynecology, the First Affiliated Hospital of Chongqing Medical University, 400016 Chongqing, China; Academician V.I. Kulakov National Medical Research Center for Obstetrics, Gynecology and Perinatology, Ministry of Health of the Russian Federation, 117997 Moscow, Russia; Department of Obstetrics, Gynecology, and Perinatal Medicine, IM Sechenov First State University, 117630 Moscow, Russia; PREIS School (The Permanent International and European School of Perinatal, Neonatal and Reproductive Medicine), 50121 Florence, Italy

**Keywords:** adverse pregnancy outcomes, birth defects, multiple micronutrient supplementation, preconception, pregnancy

## Abstract

**Objective:**

In this review we sought to determine the clinical benefits and safety of a multiple micronutrient supplement/supplementation (MMS) throughout preconception, pregnancy, and lactation in the mother and their child.

**Background:**

No guidelines for pregnancy specifically recommend supplementation with micronutrients other than folic acid and iron or continuing the use of MMS beyond the first trimester. Yet micronutrients are essential during all stages of pregnancy for healthy fetal growth and development and maternal health, with an increased intake of many micronutrients recommended during pregnancy and lactation. The MMS reviewed (Elevit, Bayer) is the most studied prenatal form of MMS, supported by 30 publications reporting studies conducted worldwide over 30 years and used by millions of women over a period of 40 years. Until now, the data have not yet been consolidated.

**Methods:**

We performed a literature search to identify published studies for trials that used MMS at any stage of the pregnancy journey.

**Results:**

Outcomes reported in 30 trials suggested that MMS improves micronutrient status, leads to a healthier reproductive environment during preconception, and can significantly reduce neural tube defects and congenital abnormalities in early pregnancy above and beyond supplementation with folic acid alone. We also found that MMS can reduce adverse pregnancy outcomes during the second and third trimesters, including miscarriage, pre-eclampsia, anemia, preterm birth, and placental insufficiency, and improve docosahexaenoic acid status. In addition, MMS improves the quality of breastmilk and reduces postpartum depression. Using MMS containing 800 μg folic acid is more effective than supplementing with 400 μg folic acid alone. Very few adverse events were reported in infants, almost all of which were considered unrelated to MMS intake. In one cohort, periconceptual MMS in children was linked to higher rates of otitis media and atopic dermatitis than placebo, but these results may be partly attributed to multiple hypothesis testing and differences in family history, respectively.

**Conclusion:**

Improving micronutrient status with MMS in women who are trying to conceive, pregnant, or breastfeeding may have beneficial effects on fertility, the integrity of the embryonic environment, development of the embryonic brain and nervous system, and the growth, development, and long-term health of the child.

## INTRODUCTION

### Good Nutrition During the First 1000 Days of Life is Essential

**Figure 1.**
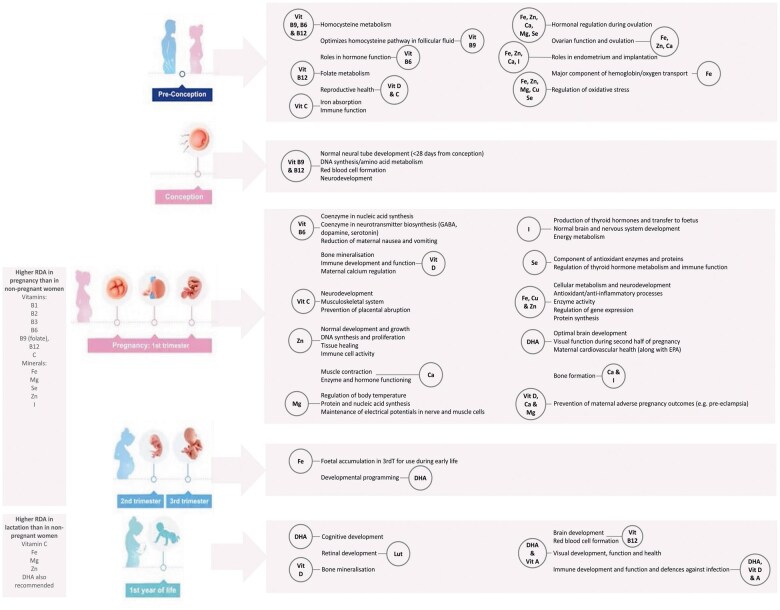
Examples of Some of the Key Roles of Micronutrients Throughout Preconception, Pregnancy, and Lactation. Abbreviations: DHA, docosahexaenoic acid; Lut, lutein; RDA, recommended dietary allowance; Vit, vitamin.

There is strong evidence to suggest that early life—conceptualized as the “first 1000 days of life” (including preconception, fetal life, and the first 2 years of infancy)—has an impact on health trajectories in later life.^1–3^ Maternal nutritional status has a major influence on fetal development and maternal health during these first 1000 days, and optimizing dietary intake is a basic step toward a healthy pregnancy.^4^^,^^5^ Epigenetics play a vital role during gestation, when the fetus experiences a critical period of plasticity—suboptimal nutrition during this time can induce epigenetic changes, specifically DNA methylation. DNA methylation patterns are a fundamental part of the embryonic development program and are necessary for gene regulation and normal cognitive function, for example—but if not precisely regulated, DNA methylation can adversely reprogram the phenotype of an individual and affect future health.^6–8^ Micronutrients in particular are essential for reproductive health, required at every stage during preconception, pregnancy, and lactation^9^^,^^10^ (Figure 1), and dietary reference intake guidelines worldwide recommend increased intake during pregnancy and lactation to address increased demands during development of the placenta and fetus.^11–15^

In the preconception period, for example, adequate nutrition and levels of micronutrients such as folate, vitamin D, and trace elements, including iron, zinc, and copper, are required for cell division, cellular membrane stability, DNA synthesis, antioxidant capability, and oocyte quality and maturation, all of which are linked to fertility and the potential for implantation.^16–21^ When conception is successful, the normal development of the embryo and fetus depends entirely on the supply of nutrients from the mother—therefore, her nutritional state is of paramount importance. Micronutrients, particularly folate and other B vitamins (B2, B6, B12), are essential for neural tube closure, which normally occurs within 4 weeks after conception; if closure is incomplete at this time, there is a risk of neural tube defects (NTDs)^22^ that can lead to serious complications such as spina bifida and anencephaly. A dose–response relationship has been demonstrated between early pregnancy red blood cell (RBC) folate levels and the risk of NTDs.^23^ Folate insufficiency also leads to high levels of homocysteine—an amino acid produced when proteins break down, and a risk factor for adverse pregnancy outcomes, including NTDs, miscarriage (the most common complication of early pregnancy), preeclampsia, preterm birth, and poor fetal growth.^16^^,^^24^^,^^25^ The risk of these and other adverse pregnancy outcomes can also be increased by anemia (which can occur when folate and vitamins B2 and B12 are insufficient) and iron-deficient anemia (IDA; which may develop if iron stores become depleted).^26^ Oxidative stress in the placenta, caused by an imbalance between oxidants and antioxidants such as vitamins A, C, and E, can contribute to first-trimester miscarriage.^27^

Micronutrients are also essential to support a growing baby during lactation.^28^ Adequate supplies of critical nutrients in breast milk are required for brain development and cognitive function, bone growth, immune functions, and the general health of the baby; micronutrient deficiencies can lead, for example, to blindness (vitamin A), rickets (vitamin D), anemia (iron), mucosal bleeding and scurvy (vitamin C), and seizures, depression, and neurological disorders (various B vitamins).^29–31^ Micronutrients have essential roles in many neuronal functions and regulate mental processes in the baby, and also in the mother, in whom the lack of certain vitamins and minerals (eg, the B vitamins, vitamin D, zinc, iron, omega-3 fatty acids) can adversely affect mental processing, leading to postnatal depression.^32^

### Micronutrient Levels May Be Insufficient During Conception and Pregnancy

Despite general awareness of the need for good nutrition during preconception and throughout pregnancy, mothers may not always find it possible to meet nutritional needs, and it may be difficult to consume the daily recommended levels of essential micronutrients, which are required in higher amounts during this time.^11^^,^^12^ For example, 69% of women of reproductive age worldwide (equivalent to 1.2 billion women) have been found to present with deficient levels of iron, zinc, and/or folate.^33^ In addition, many women may not even realize they are pregnant and will not have prepared for pregnancy, increasing their risk of micronutrient insufficiency. This “hidden hunger” can occur without any signs or symptoms in the mother, but can have long-term adverse effects on the growing baby.^34–36^ Micronutrient deficiencies are more common in developing countries, where food sources may be more limited,^34^^,^^37^ but food insecurity and food deserts are prominent global health problems.^38^

Even when relevant food sources are available, many different factors contribute to inadequate micronutrient levels in women, such as a lack of awareness of healthy food sources, dietary choices (eg, a vegan or vegetarian diet), and religious and cultural aspects (such as long, concealing clothing) or excessive use of sun blocker, which may both lead to insufficient sun exposure, and pre-existing metabolic disorders such as obesity, diabetes, and polycystic ovary syndrome.^39–41^ Furthermore, many toxic chemicals that may be ingested during nutrient intake (eg, heavy metals, persistent organic pollutants, air pollutants, alcohol, and toxins obtained from smoking, etc.) impair the normal absorption and metabolism of both macro- and micronutrients, predisposing individuals toward impaired metabolism.^42^ Thus, even in industrialized countries, where nutritious food is more available, micronutrient levels are often below recommended levels in women of child-bearing age and women who are pregnant.^43^^,^^44^ This situation is exacerbated by a lack of awareness in women themselves about the specific impact of adequate nutrition throughout the pregnancy journey, particularly the reasons why it is so important to adopt appropriate nutritional practices during this time.^45^ Yet women have a strong desire for nutritional issues to be addressed by clinicians during routine antenatal appointments,^46^ and the use of a simple nutrition checklist could help to identify women with suboptimal dietary quality in early pregnancy.^47^

### Supplementation With Multiple Micronutrients Should Continue Throughout Pregnancy and Lactation

In addition to nutritional guidance, preconceptional care guidelines generally recommend that women increase their folate intake before conception and until the end of the first trimester—a simple measure that has been proven to minimize the occurrence of NTDs.^48^ This increase in folate most often occurs in the form of a daily supplement that contains at least 400 μg folic acid to address periconceptional needs. Fortification of food with folic acid (eg, in flour) is also mandatory in 69 countries, while 47 have voluntary fortification (some countries like China do not fortify food with folate); mandatory fortification results in the highest mean plasma folate levels and the lowest prevalence of NTDs.^49^ Furthermore, it has been recommended that oral contraceptives should contain folic acid to help prevent birth defects in women who become pregnant after they stop taking birth control pills.^50^ It should be noted that approximately 8 weeks after supplementation with folic acid has ended, RBC folate concentrations decline to levels that are no longer protective against NTDs (<906 nmol/L^23^).^51^

However, several gaps remain in current periconceptional guidelines. Nutrition and micronutrients are barely mentioned in guidelines in the field of fertility, including those for in vitro fertilization (IVF). In most pregnancy guidelines (eg, those provided by the WHO^14^), 400 μg folic acid is generally recommended but only until the end of the first trimester. In addition, only a few countries, such as China, the United States, and Canada, recommend 400-800/1000 μg folic acid and concomitant multiple micronutrients until the end of the first trimester (China, United States) or before conception and throughout the whole pregnancy (Canada).^22^^,^^52^ In fact, preconceptional care guidelines on supplementation with micronutrients other than folic acid, iron, vitamin A, and iodine are uncommon—although the International Federation of Gynecology and Obstetrics (FIGO) does state that the benefits of supplementing with multiple micronutrients outweigh those observed with folic acid and iron with respect to birth and pregnancy outcomes.^15^ Almost no guideline recommends continuing supplementation with multiple micronutrients after the end of the first trimester—even though needs for some micronutrients, such as iron and calcium, are increased during the second trimester, and the levels of most micronutrients decrease during pregnancy unless supplemented.

A good rationale exists for providing women with multiple micronutrient supplements during every stage of pregnancy, not just preconception and the first trimester.^53^ As outlined in various guidelines for recommended dietary allowances,^11^^,^^12^^,^^54^ many micronutrients are required in higher amounts during conception, pregnancy, and lactation. Sufficient levels of micronutrients not only support fertility^55^ and reduce the risk of NTDs and other congenital abnormalities (CAs) during early pregnancy,^56^ but also help to improve some of the adverse outcomes associated with pregnancy, such as anemia, pre-eclampsia, and miscarriage,^57^^,^^58^ and improve the nutritional quality of breastmilk during lactation.^13^ Maternal micronutrient status in the periconceptional period and throughout pregnancy and lactation should be viewed as a continuum, not regarded as a series of separate stages with respect to micronutrient needs.^59^

In most women, a multiple micronutrient supplement/supplementation (MMS) that has been specifically tailored to the needs of pregnant women may be necessary to address increased needs during pregnancy and support the reproductive environment, with the aim of improving pregnancy and maternal outcomes. It has already been shown that many women in “high-income” countries appear to have suboptimal levels of folate (despite the well-advertised need for increased intake during pregnancy), vitamin B12, vitamin D, calcium, iodine, iron, and selenium.^43^^,^^44^ Thus, it may be necessary to recommend *continued* daily use of an MMS throughout pregnancy and lactation, to meet the increased micronutrient needs that extend beyond the first trimester.

In this review we outline some of the studies that have been performed with an MMS that was specifically designed for use during all pregnancy stages, including preconception, and has been widely used throughout the world for over 40 years (Elevit, Bayer; Table 1). This MMS brand was the first of its kind to be clinically tested for the prevention of first-occurrence NTDs and is recommended for daily use before conception and throughout pregnancy and lactation to optimize pregnancy outcomes and minimize the potential for adverse outcomes associated with pregnancy. To our knowldge, this review is the first to consolidate data from these studies, with the aim of providing a critical overview of *why* supplementing with multiple micronutrients every day—starting with the first thoughts of trying to conceive right through to breastfeeding the baby—is essential to optimize reproductive health and the health of the mother and child. Using the available evidence, it is important to determine whether there are clinical benefits associated with (1) MMS vs no supplementation, folic acid, or iron supplementation alone, and (2) continuing to use MMS into the second and third trimester and beyond into the lactation period.

**Table 1.** Overview of the vitamins and minerals in the reviewed multiple micronutrient supplement.

Vitamins^a^	Amount	Minerals^a^	Amount	Others^b^	Amount
A ^c^	770 (RE) to 2200 μg	Calcium	120–125 mg	DHA	200 mg
B1 (thiamin)	1.3-1.6 mg	Copper	0.9–1.0 mg	EPA	80 mg
B2 (riboflavin)	1.4-1.8 mg	Iodine^b^	150–225 μg	Lutein	250 μg
B3 (niacin)	12-19 mg	Iron	9–60 mg		
B5 (pantothenic acid)	5-10 mg	Magnesium	57–100 mg		
B6 (pyridoxine)	1.4-2.6 mg	Manganese	1–2 mg		
B7 (biotin)	30-200 μg	Phosphorus	125 mg		
B9 (folic acid) ^d^	400-800 μg	Selenium^b^	50–60 μg		
B12 (cobalamin)	2.6-4.0 μg	Zinc	7.5–11 mg		
C	60-100 mg				
D3	5-12.5 μg				
E ^e^	6.5-15 mg				

Abbreviations: DHA, docosahexaenoic acid; EPA, eicosapentaenoic acid; RE, retinol equivalents.

a The specific formulation adheres to local guidelines and regulations within each country.

b Only included in some formulations.

c Different forms of vitamin A were used (eg, beta carotene), depending on formulation and geography.

d Partly in the form of L-5-methyltetrahydrofolate-calcium (L-5-MTHF-Ca) in some studies (NB. 225 μg L-5-MTHF-Ca corresponds to 200 μg folic acid).

e eg, alpha tocopherol.

## METHODS

A literature search was conducted (PubMed and reference lists within published studies) for trials using the MMS (Elevit, Bayer) at any stage of the pregnancy journey. Only this specific brand of MMS was considered because it has been used globally in clinical practice for more than 4 decades. This MMS provides folic acid (800 μg) and vitamins B6 and B12, as well as additional micronutrients (Table 1) that are vital throughout every stage of pregnancy^9^^,^^10^ (Figure 1). Meta-analyses on folic acid supplementation alone have clearly demonstrated the need for this vitamin during preconception and early pregnancy,^60^^,^^61^ but mostly at a dose of around 400 μg of folate. Few studies have evaluated doses of 800 μg folic acid, and it is important to allay any potential concerns about using this dose during all pregnancy stages. There is also a need to look at data beyond folic acid, including the clinical efficacy and safety of supplementing with multiple micronutrients throughout pregnancy. This specific supplement was the first prenatal MMS to have been evaluated in placebo-controlled, randomized clinical trials (RCTs) that demonstrated its efficacy at reducing birth defects. Several clinical studies have also specifically looked at the benefits of this MMS beyond minimizing the risk of NTDs, providing insight into its impact on the risk of morning sickness, anemia, pre-eclampsia, placental insufficiency, miscarriage, and preterm delivery, as outlined below.

Because of the considerable heterogeneity between studies in terms of design, patient populations, comparator groups, and study outcomes, it was not possible to apply any synthesis methods or to examine the results via meta-analysis. Therefore, we have provided a narrative review of available studies, with data presented descriptively per pregnancy stage and study population group. We aimed to provide a concise overview of the potential benefits of supplementing with multiple micronutrients before, throughout, and beyond pregnancy.

## DISCUSSION

### Characteristics of Identified Studies

Thirty studies (8 of which reported further analyses of older studies included in our review) of women using MMS or their offspring were identified (Table 2): Hungary (*n* = 6, based on the same population; published 1992-2004); Russia (*n* = 6; 2005-2021); China (*n* = 5; 2013-2020); Germany (*n* = 5; 2009-2020); Italy (*n* = 3; 2013-2020); Japan (*n* = 2; 2021-2023); Turkey (*n* = 2, based on the same population; 2010-2011); Australia and New Zealand (*n* = 1; 2016). Four studies were in healthy, nonpregnant women (all original RCTs), 5 in women trying to conceive (1 original RCT), 5 in women undergoing IVF (2 original RCTs), 14 in women at different stages of pregnancy (2 original RCTs), and 2 postpartum (both original RCTs). Overall, the use of MMS was studied in 19  864 women. At the time the studies were conducted, and to the best of our knowledge, food fortification with folic acid was not mandatory apart from in Australia.

**Table 2.** Characteristics of All Studies Evaluating the Efficacy of the Reviewed Multiple Micronutrient Supplement.^a^

Study	Country	Design	Comparator	No. of women/offspring		Duration
				MMS	Control	
Healthy, nonpregnant women						
Brämswig et al. (2009)^63^	Germany	Double-blind, PBO-controlled RCT	PBO	21	21	16 wk
Schaefer et al. (2016)^64^	Germany	Double-blind, PBO-controlled RCT	PBO	20	20	16 wk
Pilz et al. (2017)^71^	Germany	Single-center, open RCT	MMS (400 μg folic acid) containing 200 IU vitamin D3	101	100	8 wk
Obeid et al. (2018)^69^	Germany	Single-center, open RCT (same population as Pilz 2017)	MMS containing 400 μg folic acid	101	100	8 wk
Preconception and first/second trimester						
Women trying to conceive						
Dudás et al. (1995)^78^ *^b^*	Hungary	Subanalysis of double-blind, PBO-controlled RCT	“Trace-element” PBO (Cu 1 mg, Mn 1 mg, Zn 7.5 mg, vit. C 7.5 mg)	497	513	PC until end 1stT (max. 7 mo)
Czeizel et al. (1996)^79^ *^b^*	Hungary	Subanalysis of double-blind, PBO-controlled	“Trace-element” PBO (Cu 1 mg, Mn 1 mg, Zn 7.5 mg, vit. C 7.5 mg)	3953	3952	PC until end 1stT (max. 7 mo)
Wang et al. (2017)^70^	China	Single-center RCT	Folic acid alone (400 μg)	21	17	12 wk
Kuroda et al. (2021)^66^	Japan	Consecutive case series	—	205	—	Until folate and homocysteine levels were normalized to minimize risk of neural tube defects
Radzinsky et al. (2021)^65^	Russia	Multicenter, observational intervention study	—	200	—	101 d
Women undergoing IVF						
Özkaya & Nazıroğlu (2010)^75^	Turkey	PBO-controlled RCT and age-matched controls	PBO	26	43	45 d PC
Özkaya et al. (2011)^72^	Turkey	PBO-controlled RCT with some age-matched controls (same population as Özkaya 2010)	PBO	26	43	45 d PC
Sun et al. (2013)^74^	China	PBO-controlled RCT	PBO	30	25	60 d PC
Luddi et al. (2016)^76^	Italy	Crossover trial	No supplement (1st cycle)	18	—	3 mo pre-IVF cycle
Ogawa et al. (2023)^68^	Japan	Prospective interventional study	No supplement	26	30	12 wk (PC)
Pregnant women						
Czeizel et al. (1992)^90^ *^b^*	Hungary	First 1000 pregnancies from double-blind, PBO-controlled RCT	“Trace-element” PBO (Cu 1 mg, Mn 1 mg, Zn 7.5 mg, vit. C 7.5 mg)	500	500	PC until end 1stT (max. 7 mo)
Czeizel (1994)^83^ *^b^*	Hungary	Final pooled analysis of double-blind, PBO-controlled RCT	“Trace-element” PBO (Cu 1 mg, Mn 1 mg, Zn 7.5 mg, vit. C 7.5 mg)	2471	2391	PC until end 1stT (max. 7 mo)
Czeizel et al. (2004)^84^ *^b^*	Hungary	TCT using patients from double-blind, PBO-controlled RCT recruited at 14 wk gestation	No supplement	3056	3056	PC until end 1stT (max. 7 mo)
Czeizel (2004)^85^ *^b^*	Hungary	Pooled analysis of the above RCT and TCT, plus results from the HCCSCA (same population as those in refs. ^83^ and ^84^)	RCT + TCT: offspring & HCCSCA: controls without CA	28 370	43 598	PC and 1stT
Pasman et al. (2005)^87^	Russia	Observational and retrospective analysis of case series	No supplement	43	34	3-6 mo PC and throughout gestation
Arzhanova et al. (2009)^67^	Russia	Case series	—	60	—	3 mo PC and during pregnancy
Mozgovaya et al (2011)^98^	Russia	Case series	—	60	—	1stT and 2ndT
Sun et al. (2013)^86^	China	Retrospective analysis	No supplement	2693	1502	3 mo from d of transplant
Vanderlelie et al. (2016)^100^	Aus & NZ	Prospective, longitudinal birth cohort study	No supplement or FA (400 μg)	719	1542	1stT
Lin et al. (2020)^101^	China	Retrospective analysis	Folic acid alone (400 μg)	9230	7342	1stT to 3rdT
Ou et al. (2020)^99^	China	Retrospective analysis	Folic acid alone (400 μg)	106	65	3-mo PC until end of 1stT
Second and third trimester						
Khodova & Murashko (2006)^103^	Russia	Case series	—	87	—	2ndT & 3rdT
Kurmacheva et al. (2018)^104^	Russia	Retrospective comparative analysis	No supplement/not regular users	147	229	3rdT and postpartum
Massari et al. (2020)^110^	Italy	Open RCT	No supplement	65	76	GW 13-15 until delivery
Postpartum						
Paoletti et al. (2013)^119^	Italy	RCT	Ca (500 mg) + vitamin D3 (400 IU)	424	428	3-30 d after delivery
Schaefer et al. (2020)^113^	Germany	Double-blind PBO-controlled RCT	PBO	35	35	From 4-6 wk after delivery for 12 wk
Total number of women/offspring included in these studies (NB. not all are unique studies)				**53** **311**	**65** **662**	

Abbreviations: CA, congenital abnormalities; FA, folic acid; HCCSCA, Hungarian Case-Control Surveillance of Congenital Abnormalities; PBO, placebo; PC, preconception; RCT, randomized, controlled trial; TCT, 2-cohort trial; vit, vitamin; 1stT, first trimester; 2ndT, second trimester; 3rdT, third trimester.

a Please see Table S1 for further details, including key results.

b All related to the population included the Czeizel RCT performed in Hungary.

**Figure 2.**
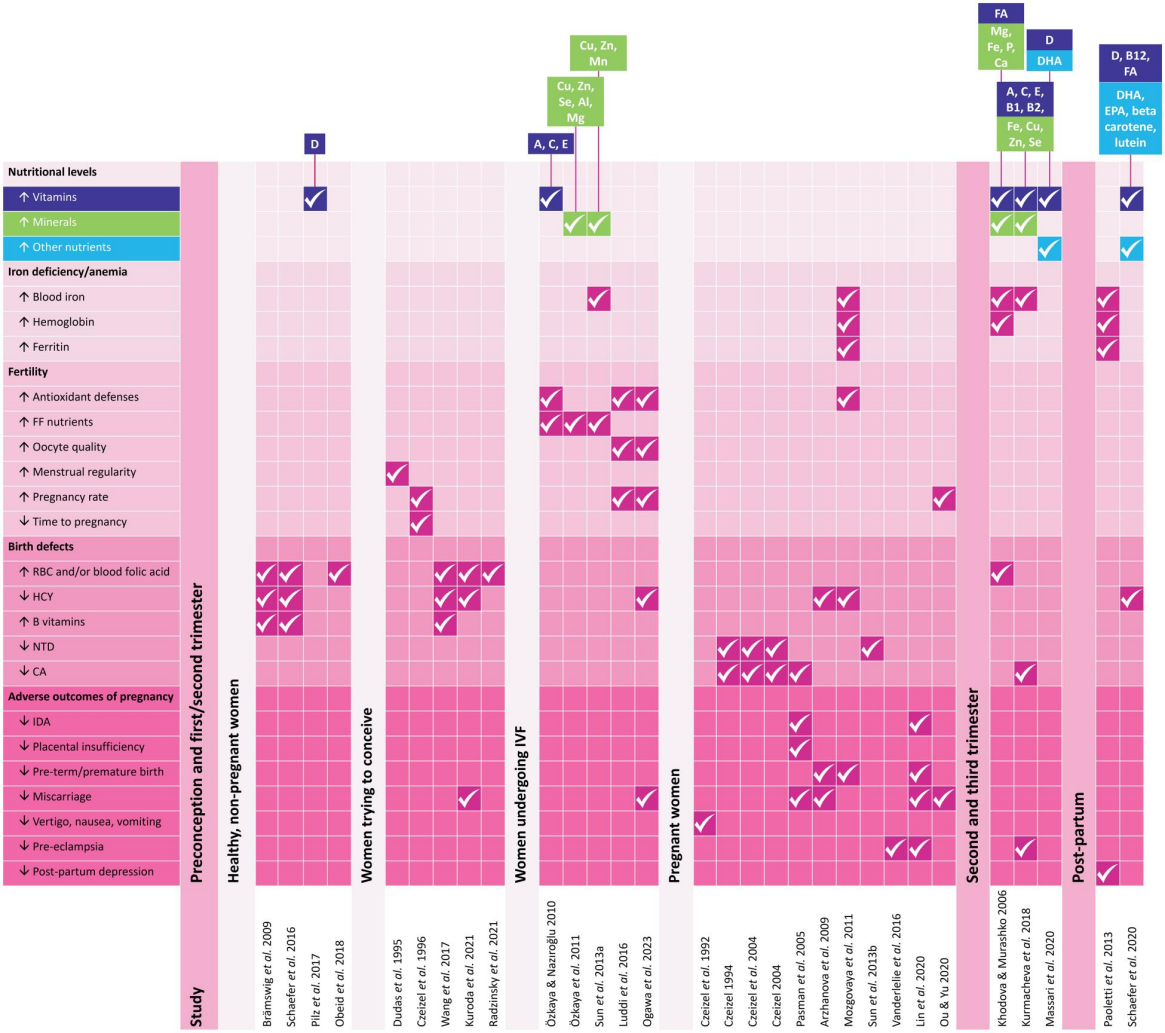
Statistically Significant Improvements in Key Efficacy Outcomes Throughout the Pregnancy Journey After Using Multiple Micronutrient Supplementation Compared to Control or Baseline (Further Details can be Found in Table S1). Abbreviations: CA, congenital abnormalities; FF, follicular fluid; HCY, homocysteine; IDA, iron-deficiency anemia; NTD, neural tube defects; RBC, red blood cell. Vitamins: FA, folic acid. Minerals: Al, aluminum; Ca, calcium; Cu, copper; Fe, iron, Mg, magnesium; Mn, manganese; P, phosphorus; Se, selenium; Zn, zinc. Others: DHA, docosahexaenoic acid; EPA, eicosapentaenoic acid.

**Figure 3.**
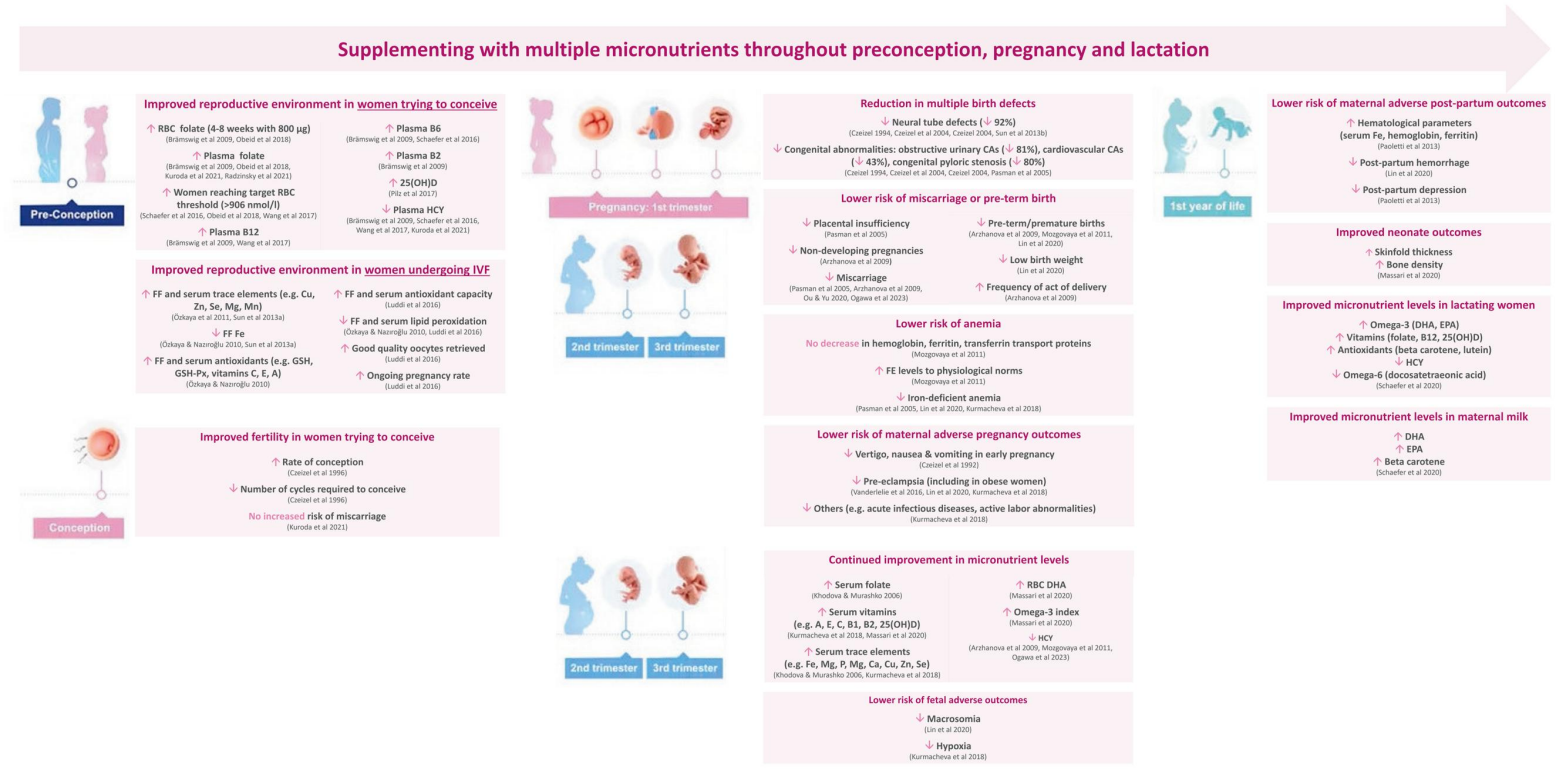
Supporting Fertility, Pregnancy, and Lactation Using a Multiple Micronutrient Supplement throughout the Pregnancy Journey—An Evidence-Based Overview. Abbreviations: 25(OH)D, 25 hydroxyvitamin D; CAs, congenital abnormalities; DHA, docosahexaenoic acid; EPA, eicosapentaenoic acid; FF, follicular fluid; HCY, homocysteine; IVF, intravenous fertilization; GSH, glutathione; Px, peroxidase; RBC, red blood cell.

Full details and key outcomes for each study can be found in Table S1. The results presented below were all statistically significant (*P* < .05), indicating improved outcomes with MMS compared with control or baseline. A summary of all studies showing significant efficacy outcomes for MMS is also illustrated in Figure 2. Figure 3 provides an evidence-based overview of the beneficial effects of MMS throughout each pregnancy stage.

### Impact of Supplementation with Multiple Micronutrients

#### Preconception

A healthy reproductive environment is essential when trying to conceive, which is supported by an adequate supply of micronutrients. For example, the metabolism of homocysteine is important for protein synthesis and to reduce inflammation within the body, a process that is dependent on folate and vitamins B6 and B12. When levels of these vitamins are low, homocysteine levels increase.^62^ Even moderate hyperhomocysteinemia is detrimental to reproductive health, resulting in reduced cell division during oogenesis or folliculogenesis, production of inflammatory cytokines, changes in nitric oxide metabolism that can affect oocyte production and fertilization, oxidative stress, cell death, and impaired methylation reactions that can reduce the production of healthy eggs.^16^ Prior to conception, it is vital that RBC levels of folate reach a threshold that is protective against NTDs (>906 nmol/L).^23^ Nucleotide synthesis and replication of DNA and RNA all rely on folate—low folate levels can result in incomplete closure of the neural tube and subsequent defects of the embryonic brain and spine during the first month of pregnancy.^22^^,^^23^ Vitamin D is also important, with immunomodulatory functions that can help to regulate inflammation that might otherwise have an adverse impact on fertility^17^; serum concentrations ≥50 nmol/L are generally regarded as adequate in most people.^12^ Furthermore, minerals and trace elements have essential roles, such as hormone regulation during the menstrual cycle, ensuring proper ovarian function and ovulation, maintaining endometrium health and receptivity for implantation, and regulating oxidative stress via antioxidant processes to prevent damage to cellular structures.^18^^,^^19^ Ovarian and uterine function is also dependent on adequate oxygen transport—a process in which iron, as an essential component of hemoglobin, plays a vital role.^18^

##### Increasing RBC folate to levels protective against NTDs and reducing homocysteine during preconception

In healthy women in Germany, administration of MMS for 16 weeks led to a significant increase in RBC folate compared with placebo, reaching protective levels within 4 weeks.^63^^,^^64^ The increase was significantly greater in women with lower baseline RBC folate levels at baseline.^63^ Plasma folate also increased compared with placebo, as did concentrations of vitamin B6^63^^,^^64^ and B2.^63^ Plasma vitamin B12 levels either increased^63^ or remained the same (compared to a decrease with placebo).^64^ In both studies, there was a significant decrease in total homocysteine with MMS, compared to no change or an increase with placebo.^63^^,^^64^ Similar results were observed in a Russian study of women planning pregnancy, in whom the mean plasma folate was 5 ng/mL at baseline; by 12 weeks, there had been a significant increase in folate levels across all women, including those with gene variants in methylenetetrahydrofolate reductase (MTHFR; an enzyme that plays a central role in folate and homocysteine metabolism) and overweight women.^65^ In infertile women undergoing fertility treatment in Japan, who had low plasma folate (<7.0 ng/mL) and high homocysteine (>13.5 nmol/L) at baseline, serum folate levels had increased to >7.0 ng/mL in all women and none had hyperhomocysteinemia after 4 weeks of MMS (regardless of the presence of additional vitamin D).^66^ Increases in folate and decreases in homocysteine to minimize the risk of NTDs were noted across MTHFR genotypes after supplementation for only 1 month. In a Russian study of women with hyperhomocysteinemia and a history of recurrent miscarriage, MMS taken for 3 months during preconception and pregnancy significantly reduced blood homocysteine levels compared with baseline.^67^ In another study of infertile women in Japan, this time in women with a history of frozen embryo transfer failure, there was a significant reduction in homocysteine after 12 weeks of MMS use during preconception compared to similar women who did not use MMS.^68^

Other studies have explored whether higher doses of folic acid—for example 800 μg instead of the usual dose 400 μg—could confer additional benefits that might offer better protection against the risk of NTDs and other CAs, for example. In healthy German women, most of whom had RBC folate levels <906 nmol/L at baseline, supplementing with an MMS containing 800 μg folic acid led to significantly higher RBC folate levels at 4 and 8 weeks than those seen with MMS containing 400 μg folic acid.^69^ Furthermore, significantly more women using the higher dose (84%) achieved protective levels after 8 weeks of supplementation compared with women using the lower dose (55%). The lower dose also led to an increase in RBC folate compared with baseline, but levels were still mostly below 906 nmol/L at 4 weeks. In a Chinese study, in women with low serum folate levels (around 7 ng/mL) at baseline who were actively trying to conceive, more women achieved target RBC folate levels at 4 weeks in the group who used MMS containing 800 μg folic acid (95.2%) compared to the group taking 400 μg folic acid alone (58.8%).^70^ In addition, there was a significant increase in vitamin B12 with the higher dose (compared to no significant change with the lower dose), accompanied by a significantly greater decrease in homocysteine from 2 weeks until the end of the study at 8 weeks. These 2 studies indicate that more than 40% of women who use 400 μg folic acid alone will be unlikely to reach RBC folate levels that are protective against NTDs within 4-8 weeks. This finding is important because most women will not realize that they are pregnant until at least 4 weeks have passed—thus, the ability to reach protective RBC folate levels in a shorter amount of time is vital to reduce the risk of NTDs.

##### Addressing increased micronutrient needs for reproductive health

Supplementing with multiple micronutrients also helps to address additional micronutrient needs during preconception. For example, insufficient vitamin D intake is widespread among women of childbearing age.^43^ In healthy women in Germany, it has been shown that MMS can increase levels of 25-hydroxyvitamin D [25(OH)D] to adequate levels (≥50 nmol/L)^71^; although a significantly greater median increase was seen with MMS containing 800 IU vs 200 IU vitamin D3 at 4 and 8 weeks, 70.4% of women using 200 IU vitamin D3 were still able to reach adequate levels after 8 weeks.

In women undergoing IVF, severe dietary deficiencies of trace elements such as copper, selenium, and zinc are commonly observed, along with higher follicular fluid (FF) levels of iron^72^ that are detrimental to the reproductive environment.^73^ It has been demonstrated that MMS had a normalizing effect on trace element levels in women undergoing IVF in Turkey and China, with significant increases in FF levels of copper, zinc,^72^^,^^74^ and selenium^72^ with MMS taken for 45^72^ or 60^74^ days before conception compared with placebo. There was also a corresponding increase in serum levels of copper, zinc,^72^^,^^74^ selenium,^72^ and manganese,^74^ as well as a significant reduction of iron levels in FF.^72^^,^^74^

##### Reducing oxidative damage

Increased lipid peroxidation and decreased levels of antioxidants have been recorded in women undergoing IVF in Turkey compared with healthy controls.^75^ When MMS was administered for 45 days prior to conception in women undergoing IVF, significant increases in vitamins A and C and the peroxidase enzyme of the antioxidant glutathione (GSH) were observed in FF, along with significant increases in serum vitamins C and E and GSH.^75^ There were also corresponding significant decreases in FF and serum levels of lipid peroxidation. When MMS was administered for 3 months prior to a second IVF cycle in Italy, the total antioxidant capacity in FF and serum increased significantly compared to no treatment in the first IVF cycle.^76^ This resulted in significant protection from oxidative damage in FF and serum proteins. It was concluded that MMS, when started 3 months before the IVF cycle, protected the follicular microenvironment from oxidative stress,^76^ which could be linked to improved chances of conception.^77^

##### Improving fertility

Improving reproductive health by using MMS during preconception, presumably via mechanisms such as those outlined above, could help to improve fertility. A recent analysis of infertile women with a history of IVF or intracytoplasmic sperm injection failure in Japan found a significant inverse relationship between homocysteine levels and serum 25(OH)D, as well as a correlation between high homocysteine levels and a lower rate of fertilization.^68^ Thus, the findings that MMS decrease homocysteine levels^63^^,^^64^^,^^66^^,^^70^ and increase levels of serum 25(OH)D,^71^ combined with its normalizing effect on micronutrient levels, should have a positive impact on fertility.

Although rarely evaluated in studies, use of MMS throughout preconception in Hungary led to more regular menstrual cycles in healthy, well-nourished women trying to conceive compared with placebo—mainly in women with irregular cycles.^78^ In addition, a significantly higher rate of conception was observed in the same population of women compared with placebo, representing a 5% increase in fertility.^79^ The time required to conceive was also significantly shorter with MMS, which required fewer menstrual cycles to achieve conception than placebo. It was concluded that the mechanism for increased fertility may be related to more regular menstrual cycles because of improved hormonal status.^79^ Furthermore, in women who used MMS for 3 months before their IVF cycle in Italy, significantly fewer poor quality oocytes were retrieved and the subsequent ongoing pregnancy rate was significantly higher compared to the cycle with no treatment.^76^

#### First/Second Trimester

It is essential to continue taking folic acid throughout the first trimester to ensure that RBC folate levels remain above 906 nmol/L and to reduce the risk of hyperhomocysteinemia that continues to be detrimental throughout pregnancy, causing endothelial dysfunction that can result in placental abruption, fetal growth restriction, pre-eclampsia, and recurrent miscarriage.^24^ Furthermore, it likely that the increased intake recommended for other micronutrients during pregnancy (ie, vitamins B1, B2, B3, B6, B12, and C, iron, magnesium, selenium, zinc, and iodine^11^^,^^12^^,^^80–82^) can only be fully met by using a supplement containing multiple micronutrients in addition to folic acid—dietary intakes of folate; vitamins B2, B6, B12, and D; and calcium, iron, and iodine, for example, have been found to be insufficient to a variable degree among pregnant women.^43^

##### Lowering the risk of NTDs and other CAs during pregnancy

Analyses of results from thousands of pregnant women in Hungary have confirmed the protective effect of folic acid against the risk of NTDs and other CAs. In the original RCT, MMS was compared against a placebo containing trace elements (copper, manganese, zinc, and vitamin C), both taken throughout preconception until the end of the first trimester. In the final pooled analysis, no NTDs were reported in the MMS group compared to 6 in the placebo group, and the difference was found to be significant.^83^ In addition, the rate of CAs was significantly lower with MMS compared with placebo, even after exclusion of the 6 NTDs in the placebo group. The reduction in CAs was mainly explained by the reduction in the rates of congenital cardiovascular malformations, urinary system defects, and congenital hypertrophic pyloric stenosis. The author concluded that the protective effect against CAs could be related to the intake of folic acid plus other vitamins.^83^ In a later 2-cohort trial, women from a Hungarian RCT were recruited at 14 weeks of gestation and matched against pregnant controls who had not used any micronutrient supplement, to further evaluate the impact of MMS on NTDs and other CAs.^84^ It was observed that the risk of NTDs was significantly lower with MMS than in controls without MMS, with no increased risk in women with a family history of NTDs. Furthermore, MMS led to a significantly lower risk of cardiovascular CAs (mainly due to fewer ventricular septal defects with MMS) and stenosis/atresia of the pelvic ureteric junction. These results confirmed that MMS had protective effects against NTDs and led to the primary prevention of some major structural defects, even in women at high risk of these adverse pregnancy outcomes.^84^ Results in women from both the Hungarian RCT^83^ and the 2-cohort trial^84^ were then compared against data for offspring included in the Hungarian Case–Control Surveillance of Congenital Abnormalities (HCCSCA).^85^ In the intervention trials,^83^^,^^84^ MMS use vs no supplement significantly reduced the risk of NTDs (odds ratio [OR], 0.08), obstructive urinary CAs (OR, 0.19), cardiovascular CAs (OR, 0.57), and congenital pyloric stenosis (OR, 0.20). The author concluded from these results that MMS prevented 92% of NTDs.^85^ Results from the HCCSCA demonstrated that folic acid alone also prevented NTDs (first month: OR, 0.68), as well as posterior cleft palate (first month: OR, 0.50), cardiovascular CAs (second month: OR, 0.75), rectal/anal atresia/stenosis (second month: OR, 0.39), and new candidate CA (hypospadias, poly/syndactyly, and multiple CA, especially when taken during the first month). However, MMS was found to be more effective than folic acid alone at reducing the risk of both NTDs and CAs. Thus, daily use of an MMS that includes 400-800 μg folic acid was recommended with a healthy diet and lifestyle in women to reduce the risk of NTDs and some CAs.^85^

In a later study of women who became pregnant after IVF in China, MMS use for 3 months from the day of transplant resulted in no NTDs compared with 6 cases in similar women who did not use any supplement.^86^ There was also a significant reduction in functional CAs requiring correction in women with hyperandrogenism in Russia who used MMS for 3-6 months during preconception and throughout pregnancy compared to no supplementation.^87^

##### Lowering the risk of adverse pregnancy outcomes

Morning sickness (nausea and vomiting) is common during early pregnancy, affecting most women in their first trimester,^88^ with many women trying often unusual remedies in an attempt to alleviate the condition. It is possible that insufficient levels of micronutrients, particularly vitamin B6, may contribute to morning sickness.^89^ Early analysis of women in the Hungarian RCT found that using MMS compared to the trace element placebo from preconception until the end of the first trimester significantly lowered the rate of vertigo, nausea, and vomiting both in early pregnancy and at the end of the first trimester.^90^ It was thought that this effect was likely due to the combined effect of micronutrients that optimized the nutritional status and metabolism in the expectant mothers.

Pregnancy can also result in outcomes with more serious consequences, such as the risk of miscarriage and preterm delivery (affecting almost 8%-24%^91^ and 10% of all pregnancies,^92^ respectively), pre-eclampsia, anemia, and placental insufficiency. Many factors can increase these risks. For example, hyperhomocysteinemia (a metabolic consequence of folate insufficiency) increases the risk of complications related to the placenta and has been associated with recurrent miscarriage, preterm birth, and pre-eclampsia.^24^ Hypertensive disorders of pregnancy are one of the main causes of maternal deaths worldwide (after hemorrhage),^93^ and are issues that need to be addressed. The causes of pre-eclampsia, which has a complex pathophysiology, are multifactorial—but it can be exacerbated by certain deficiencies of nutrients, such as vitamins D, C, and E and minerals calcium, iron, and zinc.^94^^,^^95^ An imbalance between oxidants and antioxidants and the resulting oxidative stress in the placenta is also a major risk factor contributing to first-trimester miscarriage.^27^ It is possible that folic acid supplementation or MMS have the potential to reduce the risk of miscarriage or stillbirth.^96^^,^^97^

In pregnant women hospitalized primarily for threatened miscarriage in Russia, a 2-fold decrease in homocysteine was observed in those who used MMS during the first and second trimester compared with baseline.^98^ In addition, MMS normalized total antioxidant capacity and increased the total anti-radical activity in the second trimester. No increase in coagulative potential was observed in most of these women, and no premature or operative deliveries occurred. In the Russian study in women with hyperhomocysteinemia and a history of recurrent miscarriage who took MMS for 3 months during preconception and pregnancy,^67^ there was a significant reduction in the incidence of spontaneous miscarriage and nondeveloping pregnancies—compared with previous pregnancies, the act of delivery increased by 2.5 times, term birth increased by 4 times, and preterm birth decreased by 6.6 times.

In infertile women with MTHFR genotypes undergoing fertility treatment in Japan, who had been supplemented with MMS (with or without additional vitamin D) for 6 months, it was observed that no women had hyperhomocysteinemia after 1 month of MMS use.^66^ Furthermore, there was no significant difference in the rate of miscarriage (total ≤8.7%) associated with MTHFR genotypes in pregnant women. In another study of infertile women in Japan, this time with a history of frozen embryo transfer failure, there was significant decrease in the rate of miscarriage in pregnant women who had used MMS for 12 weeks compared to controls (women recruited using the same criteria, but who did not use MMS).^68^

Other studies have also shown a beneficial effect of MMS on the rate of miscarriage. In hyperandrogenic women in Russia, there was a significant reduction in the rate of threatened miscarriage in pregnant women who used MMS compared to those who did not.^87^ In a Chinese study, pregnant women with a history of unexplained recurrent miscarriage were supplemented with MMS for 3 months before conception and until the end of the first trimester (plus acetylsalicylic acid 100 mg for 3 months before conception then 75 mg and prednisone 5 mg throughout).^99^ In these women, there was a significantly higher rate of successful treatment (defined as a 12-week pregnancy with an obvious embryo and embryonic heart revealed by ultrasound examination, nuchal translucency thickness <0.25 cm, size consistent with gestational age, and no early malformation) than in women who used folic acid alone (400 μg) during the same period.

Using MMS during pregnancy has also been shown to have a beneficial effect on the risk of pre-eclampsia. In pregnant women in Russia hospitalized primarily for threatened miscarriage, in whom there had been a significant reduction in homocysteine compared with baseline, mild pre-eclampsia was still present after using MMS during the first and second trimester—however, the rate (5.5%) was lower than normally seen in the general population.^98^ In pregnant women who had been stratified by weight in Australia and New Zealand, the overall occurrence of pre-eclampsia was significantly lower in pregnant women who used MMS during the first trimester than in the control group who used either folic acid (400 μg) alone or no supplement.^100^ After adjustment, the risk of pre-eclampsia remained significantly lower in pregnant women who used MMS compared with those who used no supplement (OR, 0.33), and in overweight or obese pregnant women who used MMS (OR, 0.48); the protective effect of MMS increased with increasing body mass index (overweight women, OR, 0.45; obese women, OR, 0.38). In an analysis in China, a significant reduction in pre-eclampsia was observed in pregnant women who used MMS in early pregnancy or throughout pregnancy compared to women who used only folic acid (400 μg) alone.^101^

The increased systemic requirements for iron during pregnancy can deplete iron stores within the body and increase the risk of IDA, unless those stores are replenished; deficiencies in folate and vitamin B2 (water-soluble vitamins that cannot be stored in the body) and vitamin B12 also contribute to the risk of anemia.^26^ Anemia is a common problem throughout the world, even in developed countries (where intake of all of these micronutrients is often inadequate^43^) and is associated with adverse impacts on maternal and fetal health.^26^

In pregnant women hospitalized primarily for threatened miscarriage in Russia, use of MMS (containing 60 mg iron) during the first and second trimester had an effective preventive role in IDA.^98^ Compared to baseline, there was no decrease in hemoglobin by the second trimester and a significant increase in serum iron levels to normal physiological values, while the levels of ferritin and transferrin transport proteins did not change significantly. An analysis performed in China has also shown that using MMS (containing 60 mg iron) in early pregnancy or throughout pregnancy significantly reduced the incidence of IDA compared to folic acid (400 μg) alone.^101^ Significant reductions in the incidence of fetal macrosomia and postpartum hemorrhage were also observed when MMS was taken during the whole pregnancy, as well as a significantly lower risk of intrahepatic cholestasis of pregnancy, premature birth, postpartum hemorrhage, and low birth weight when MMS was taken during early pregnancy.

Reducing the risk of anemia during pregnancy could also have a beneficial effect on the risk of placental insufficiency.^102^ In both healthy pregnant women and those with a hyperandrogenic condition (a high-risk group of women with respect to premature deliveries and placental insufficiency) in Russia, there was a significantly lower rate of IDA and a lower risk of placental insufficiency when MMS (containing 60 mg iron) was used for 3-6 months preconception and throughout gestation compared to similar pregnant women who did not use a supplement.^87^

#### Second and Third Trimester

The benefits of continued supplementation with multiple micronutrients have been found to extend throughout the second and third trimester, particularly in women at higher risk of adverse pregnancy outcomes, and may include beneficial effects on maternal health and support during fetal growth. For example, an analysis of pregnant women with anemia, pre-eclampsia, or thyroid disorders in Russia determined that MMS taken in the second and third trimester addressed deficiencies in folic acid, iron, magnesium, and other trace substances; had a beneficial effect on the course of pregnancy; and decreased the rate of obstetric complications.^103^ In anemic pregnant women (who had low folate, hemoglobin, erythrocyte, and hematocrit levels at baseline), MMS (which included 60 mg of iron) increased folate levels to physiological levels and significantly increased magnesium and iron.^103^ In pregnant women with pre-eclampsia, MMS significantly increased folic acid, phosphorous, magnesium, calcium, and iron levels by the end of pregnancy.^103^ In all pregnant women with anemia or pre-eclampsia, there was a significant increase in hemoglobin, erythrocytes, hematocrit, and serum iron by the end of pregnancy.^103^ In pregnant women with thyroid disorders, MMS (which did not contain iodine) significantly increased folic acid, phosphorous, and iron.^103^ It was concluded that the MMS used was effective for the prevention and combination treatment of mild and moderate anemia and pre-eclampsia and enabled individual and appropriate hormonal correlation with iodine drugs in women with thyroid disorders.^103^

In another Russian study in pregnant women who used MMS from the early stages of pregnancy and continued throughout all trimesters and postpartum, it was determined that the subsequent regulation of micronutrient intake during pregnancy resulted in improvements in obstetric and perinatal outcomes.^104^ It was observed that in women not using MMS regularly or at all during pregnancy, multiple hypovitaminosis was common and was significantly associated with obstetric and perinatal complications, including maternal anemia, chronic fetal hypoxia, acute maternal infectious diseases during pregnancy, pathological delivery in mothers, adaptation disorders, perinatal damage in the central nervous system (CNS), and acute infectious diseases in neonates.^104^ However, regular use of MMS (containing 60 mg of iron plus additional potassium iodide, 250 μg) significantly increased blood levels of iron, copper, zinc, and selenium, as well as vitamins A, E, C, B1, and B2 in the third trimester. In addition, MMS significantly reduced the rates of pre-eclampsia, anemia, chronic fetal hypoxia, acute maternal infectious diseases, and active labor abnormalities in mothers. Furthermore, there was a significant reduction in disharmonic physical development, adaptation disorders, perinatal damage in the CNS, and acute infectious diseases in neonates.^104^ These results confirmed the value and expediency of continuous micronutrient supplementation throughout pregnancy.

Docosahexaenoic acid (DHA) is an omega-3 fatty acid that plays a key role in the development of the fetal brain and begins to rapidly accumulate after the first trimester, when the neural tube has closed and gray matter begins to form.^105^^,^^106^ However, the body is not efficient at producing DHA,^107^ and the levels of DHA available to the fetus are governed by the maternal diet.^105^ Furthermore, low dietary intake and blood levels of DHA and eicosapentaenoic acid (EPA; another omega-3 fatty acid) are associated with an increased risk of preterm birth.^108^ Thus, supplementation with MMS that contains DHA could be beneficial. An additional intake of DHA >100-200 mg/d is recommended in pregnant women, preferably beginning in the second trimester of pregnancy (no later than 20 weeks of gestation) and continuing until childbirth.^109^ It has been shown in an RCT that in pregnant women in Italy who used MMS (400 μg folate) plus DHA (200 mg) from gestational week 13 to 15, there was a significantly greater increase in RBC DHA levels than in women who did not use any supplement; the increases in RBC DHA were greater in women with lower levels at baseline, and all women had reached the RBC DHA threshold (5%) by the end of the study.^110^ In addition, there was a significantly better RBC DHA/total fatty acids ratio and omega-3 index and higher 25(OH)D levels with MMS compared to no treatment. In the infants born to mothers who used MMS, there was a significantly thicker skinfold thickness and greater bone density compared to infants whose mothers did not use a supplement.^110^ The authors concluded that using MMS plus DHA in pregnant women in industrialized countries can complement dietary intake and significantly improve maternal DHA and vitamin D status—an important finding considering the essential roles of DHA and vitamin D during pregnancy.^110^

#### Postpartum

Exclusive breastfeeding is the recommended feeding method for the first 6 months of an infant’s life. Thus, the increased need for micronutrients continues into the lactation period, to support maternal health and neonatal growth and development.^11^^,^^12^ However, it has been shown that women in Russia who were not regularly using MMS or were not using MMS at all during pregnancy had low concentrations (ie, 50%-60% below normal values) of vitamins A, B1, B2, and C and beta-carotenoids, iodine, Fe, Zn, and Se in the breast milk of lactating women.^104^ In addition, accumulation of DHA in the infant brain continues for up to 2 years,^111^ so a constant maternal intake is necessary to support breastfed babies. Also needed is a regular supply of lutein—a carotenoid that preferentially accumulates in the infant brain and has an impact on retinal function.^112^ In lactating women with no overt nutritional deficiencies in Germany, macro- and micronutrient intake from food alone was often shown to be insufficient.^113^ Therefore, it is important to continue using MMS during lactation to address the increased micronutrient needs, because the nutritional status of the mother determines the quality of breastmilk.

In a German RCT of healthy lactating women from high-income countries, use of MMS (containing 500 μg folic acid plus 200 mg DHA and 250 μg lutein) during the lactation period led to significant increases in maternal milk levels of DHA, EPA, and beta-carotene, and maternal blood levels of DHA, EPA, 25(OH)D, folate, vitamin B12, lutein, and beta-carotene—all of which decreased with placebo.^113^ There was also a significant decrease in maternal blood levels of homocysteine with MMS compared to an increase with placebo; docosatetraenoic acid (an omega-6 fatty acid that can have proinflammatory effects on accumulation^114^) decreased with MMS and placebo, but to a significantly greater extent in the MMS group.^113^

Insufficient levels of micronutrients, particularly the B vitamins, vitamin D, and trace minerals, have been implicated in the etiology of postpartum depression—a relatively common and often severe mood disorder that can develop in women after childbirth.^115^ Low maternal serum vitamin D levels also increase the risk of depression during pregnancy—thought to affect 10%-20% of women in high-income countries^116^—with a subsequent increased risk of postpartum depression.^117^ Anemia is also another risk factor for maternal depression.^118^ An RCT in Italy of healthy women without risk factors for depression compared the use of MMS 3-30 days after delivery to supplementation with calcium (500 mg) plus vitamin D3 (400 IU).^119^ Significant and comparable increases in anemia-related indicators were observed in both supplemented groups, including blood iron, hemoglobin, and ferritin. However, there was a significantly greater improvement in postnatal depression (ie, decrease in Edinburgh Depression Postnatal scale [EPDS] score) with MMS vs the control supplement, which was particularly evident in women with a basal EPDS score <12.^119^ It was concluded that MMS favorably modulated brain functions antagonizing the evolution to postpartum depression.

### Safety of MMS

In total, this review included 10 studies that reported the occurrence of any adverse effects in women (5 in healthy, nonpregnant women^63–65^^,^^69^^,^^71^; 4 in pregnant women^67^^,^^90^^,^^98^^,^^110^; 1 in lactating women^113^). Two studies specifically looked at any potential safety issues in infants after maternal supplementation from preconception until the end of the first trimester,^120^^,^^121^ while any adverse events reported in offspring were also outlined in another 2 studies.^110^^,^^113^ Full details can be found in Table S2. As outlined below, these studies indicate that this MMS (which has been used by millions of women worldwide) throughout preconception, pregnancy, and lactation has a good safety and tolerability profile, with no long-lasting or serious adverse effects on maternal health or on the developing fetus.

#### Safety Profile in Women

Overall, the use of MMS during preconception and throughout pregnancy has been found to be well tolerated in women (*n* = 1263), with no significant differences in adverse events compared with no supplementation,^110^ placebo,^63^^,^^64^^,^^90^^,^^113^ or supplementation with a lower dose of folic acid (400 μg).^69^^,^^71^ The most commonly reported treatment-related adverse events were associated with gastrointestinal intolerability.^64^^,^^98^^,^^110^ Only 1 study, conducted in Germany, reported that gastrointestinal complaints, including diarrhea, nausea, and flatulence, occurred more often with MMS than placebo.^64^ No adverse events led to treatment discontinuation, and no severe or serious adverse events were reported, including death, with the exception of 1 study in Italy, in which the death was unrelated to treatment.^110^ In the studies that evaluated laboratory parameters for safety, including kidney and liver function, parameters were within normal ranges.^110^ Some studies have evaluated whether there is an association between micronutrient intake and gestational diabetes (a common complication of pregnancy), with inconclusive results.^122^^,^^123^ In the clinical trials included in this review, there were no reports of a higher risk of gestational diabetes mellitus or macrosomia after intake of 800 μg folic acid throughout preconception, pregnancy, and lactation.

#### Safety Profile in Infants

In infants, 1 study in Germany reported that there were no treatment-related adverse events with MMS,^113^ while another study in Italy stated that 1 woman had a treatment-emergent adverse event pertinent to the fetus/child that led to permanent discontinuation; however, whether the event was suspected to be treatment related was not reported.^110^ There has been some concern that excessive supplementation with folic acid could cause epigenetic effects and long-term health problems, including the risk of neonatal death, via modulation of DNA methylation. However, these concerns were not reflected in the Hungarian studies that evaluated postnatal somatic and mental development after periconceptional MMS either in the short term (*n* = 1809 infants)^120^ or over a longer period of 2 or 6 years (*n* = 323 infants).^121^ No significant differences between infants after maternal MMS or placebo were found in the short term in terms of mortality, overall rates of serious or chronic disorders, somatic development (body weight and length, head circumference), mental and behavioral development, and social skill quotient.^120^ There was a significantly higher rate of atopic dermatitis in MMS infants, but 4 of the 15 children had a parent with atopic dermatitis.^120^ There was also a significantly higher rate of asthma and wheezy bronchitis in MMS infants; once again, 6 of the 26 children affected had a positive family history, in contrast to none of the maternal placebo infants. As this study did not include a sensitivity analysis to determine whether the higher rate with MMS remained significant after removing children with a family history, it may be advisable to evaluate any allergenic potential in future studies. Long-term follow-up over 2 and 6 years confirmed that there were no adverse effects of periconceptional MMS on the long-term postnatal somatic and mental development of children.^121^ There were no significant differences between infants after maternal MMS or placebo in terms of overall rate of allergies (including atopic dermatitis), anthropometric data, ophthalmological and audiological examinations, developmental variables, and intelligence or development quotients. However, there was a significantly higher rate of otitis media after maternal MMS, which was borderline significant at 2 years and slightly more significant at 6 years; it was suggested that this result might have been the consequence of chance due to multiple comparisons, but required further investigation. There were no reports of teratogenic effects (which may have been a concern with long-term use of a supplement containing vitamin A).

## CONCLUSION

Good nutrition during the first 1000 days of life is vital for maternal health, normal development of the fetal body and brain, and long-term health of the child. An inadequate supply of micronutrients—whether when trying to conceive, during pregnancy, or when breastfeeding—has adverse effects on fertility, integrity of the embryonic environment, development of the embryonic brain and nervous system, and the growth, development, and long-term health of the child.

Yet despite awareness of the importance of maternal nutrition, micronutrient status is often inadequate in women of child-bearing age and pregnant women, even in industrialized countries. Supplementing with folic acid is already widely recommended to prevent NTDs—but supplementation with multiple micronutrients that have been specifically tailored for pregnancy is also important for prevention of multiple birth defects and to address the “hidden hunger” and fully support maternal and fetal health. Supplementation with MMS may also be necessary in women struggling to conceive and can help to improve the reproductive environment—a concept that to our knowledge is not currently discussed in fertility guidelines.

The MMS reviewed here (Elevit) has a strong heritage, having been used by millions of women for more than 40 years. To our knowledge, this MMS brand is the most studied prenatal multiple micronutrient, supported by 30 publications conducted in many countries worldwide over 30 years (from 1992 to 2023). These studies demonstrate that supplementing with multiple micronutrients that are required throughout every stage of pregnancy (Figure 1) has beneficial effects (Figure 3), as outlined below.

During preconception, MMS increased micronutrient levels (iron, vitamin D, copper, zinc, selenium, manganese) in blood and/or FF (apart from a decrease of iron in FF) and increased antioxidant levels (vitamins A, C, and E, and GSH), thereby reducing lipid peroxidation and oxidative stress. These actions led to a healthier reproductive environment that supported and improved fertility—including women who were undergoing IVF, in whom MMS normalized micronutrient levels in serum and FF and homocysteine concentrations, and reduced the risk of oxidative stress. In addition, MMS with 800 μg folic acid increased RBC folate to levels protective against NTDs (>906 nmol/L) in 4 weeks; increased levels of vitamins B2, B6, and B12; and lowered the risk of hyperhomocysteinemia in all women, including those with MTHFR genotype mutations that can affect their ability to process folic acid.

In early pregnancy, MMS with 800 μg folic acid plus vitamin B6 and B12 prevented 92% of NTDs, with a significantly lower risk even in women with a family history of NTDs. There was also a significant reduction in other CAs, ie, obstructive urinary CAs (by 81%), cardiovascular CAs (43%), and congenital pyloric stenosis (80%). For both NTDs and CAs, MMS was more effective than supplementing with folic acid alone. It should be noted that supplementing with inositol may also reduce the risk of NTDs^124^; however, further research is required to support this pilot trial. Furthermore, MMS reduced the risk of nausea, vomiting, and miscarriage (attributed to the combined effect of micronutrients that optimized the nutritional status and metabolism). Early use of MMS also reduced the risk of pre-eclampsia, especially in overweight or obese women, while using MMS that contained 60 mg iron from preconception or the first trimester reduced the risk of anemia later in the pregnancy journey.

During the second and third trimester, MMS continued to normalize micronutrient levels (ie, folic acid, iron, calcium, magnesium, copper, zinc, and vitamins A, E, C, B1, and B2) and thereby reduced adverse pregnancy outcomes. These included miscarriage (via a reduction in hyperhomocysteinemia; the reduction was also seen across women with various MTHFR genotypes and those at increased risk of miscarriage), pre-eclampsia, anemia (including IDA, via improvements in iron status and anemia indicators such as hemoglobin, hematocrit, ferritin, and transferrin transport proteins), preterm births, and placental insufficiency (in healthy women and those at high risk). Maternal DHA and vitamin D status also improved.

Postpartum, MMS improved the quality of breast milk (ie, increased maternal milk levels of omega-3 fatty acids [DHA, EPA] and beta-carotene, increased maternal blood levels of DHA, EPA, 25(OH)D, folate, vitamin B12, lutein, and beta-carotene, decreased levels of the omega-6 fatty acid docosatetraenoic acid), and reduced postpartum depression by favorably modulating brain functions (likely via normalizing levels of various micronutrients).

The MMS reviewed, which contains 800 μg folic acid, was significantly more effective than 400 μg folic acid alone in terms of (1) quickly increasing RBC folate to protective levels at 4 weeks (vs 400 μg folic acid alone *and* MMS containing 400 μg folic acid), significantly reducing homocysteine levels from 2 weeks, and increasing vitamin B12 levels; (2) increasing the chance of a healthy pregnancy at 12 weeks in women at risk of miscarriage; (3) significantly reducing the risk of pre-eclampsia during the first trimester and throughout pregnancy; and (4) significantly reducing the risk of IDA and subsequent adverse pregnancy outcomes (for MMS containing 60 mg iron—which is within WHO recommendations of 30-60 mg in areas with a high prevalence of anemia^14^).

Intake of MMS throughout pregnancy did not result in a higher frequency of adverse events or complications compared to no supplement, placebo, or 400 μg folic acid, apart from some instances of increased mild gastrointestinal complaints compared with placebo. Significantly higher rates of atopic dermatitis, asthma, and wheezy bronchitis were noted with MMS in children, albeit including those with a family history of such conditions; thus, further investigation into the allergenic potential of MMS may be required, as well as for the impact of MMS on the risk of otitis media in young children. None of the clinical studies included reported food allergies after MMS, or the occurrence of autism spectrum disorder (ASD) in the study in older children.^121^ In fact, studies that have specifically evaluated the impact of exposure to folic acid before and during pregnancy found that the risk of ASD decreased in infants with maternal folic acid supplementation compared to infants without maternal nutritional supplementation.^125–128^ These findings do not support assertions that higher levels of folic acid, particularly in countries where food is fortified with folic acid, could lead to increased levels of unmetabolized folic acid and thus a higher risk of these conditions.^129^ There were no reports of an adverse impact on liver or kidney function.

Women’s nutrition and health during the “first 1000 days of life” play an important role in the intergenerational transmission of human health capital, with the potential to improve future health, happiness, longevity, and economic progress. The health of the next generation will benefit through reduced risk of stunting, obesity, chronic noncommunicable diseases, and improved cognitive and behavioral development. Yet a holistic approach to nutrition in pregnancy is currently lacking. The usual advice to take folic acid until week 12 may be *insufficient* for a healthy pregnancy. Gynecologists, midwives, and mothers-to-be are generally unaware of current recommendations, and many obstetricians and midwives do not provide advice on nutrition. It has been advised by FIGO that obstetricians and midwives have an important role to play in optimizing nutrition for the mother and baby,^80^ and FIGO also recommends MMS for pregnant women who do not attain an adequate diet. Well-nourished women may not need MMS to satisfy the daily requirements—but in the absence of a careful evaluation by a nutritionist, it is prudent to recommend an MMS. Ideally, nutritional monitoring and laboratory tests could in the future help to personalize supplementation and ensure that the necessary nutrients are provided safely in doses appropriate to each woman considering pregnancy. In the meantime, the specific content of MMS formulations may evolve (for example, it is possible that higher levels of vitamin D could lead to more beneficial results)—but this very much depends on current evidence and local guidelines and regulations.

In this review we have shown that using MMS throughout the entire pregnancy journey (from preconception to pregnancy and breastfeeding), rather than preconception and the first trimester alone, can support maternal nutritional needs and the healthy development of the baby. However, there is a need for more healthcare providers to recommend the use of MMS during every stage of pregnancy, and help increase the use of MMS worldwide. It is imperative that this simple and proven intervention—used alongside a healthy, balanced diet where possible, and under the guidance of a healthcare professional (who should consider the individual needs of the woman and monitor any pre-existing conditions)—becomes a common and accepted practice in women planning a pregnancy and that such a supplement is easily available to all women worldwide to help optimize pregnancy outcomes. Currently, no guidelines for pregnancy specifically recommend supplementing with vitamins and minerals other than folic acid and iron, or continuing use beyond the first trimester. This is a situation that, based on the evidence, could be addressed in the future.

## Supplementary Material

nuaf079_Supplementary_Data
