# Supplementary material for: Clinical Benefits and Safety of Multiple Micronutrient Supplementation During Preconception, Pregnancy, and Lactation: A Review
Source: Nutr Rev. 2025 Jul 14;83(12):2352–71. doi: 10.1093/nutrit/nuaf079 (PMC12603366; doi:10.1093/nutrit/nuaf079)
Supplement: nuaf079_Supplementary_Data [file nuaf079_supplementary_data.docx]

**Supplementary Table 1.** Efficacy of supplementation with multiple micronutrients *^a^* throughout preconception, pregnancy and lactation.

| **Study (country)** | **Design** | **Study groups (N)** | | **Treatment** | | | **Key results** | **Conclusions** |
| --- | --- | --- | --- | --- | --- | --- | --- | --- |
|  |  | **MMS** | **Control(s)** | **MMS: folate (μg)** | **Control(s)** | **Duration** |  |  |
| ***Outcomes preconception***  *Healthy non-pregnant women* | | | | | | | | |
| [Brämswig *et al.* 2009](#_ENREF_2)  (Germany) | Double-blind, PBO-controlled RCT | Healthy females with RBC folate <800  nmol/l (21) | Healthy females with RBC folate <800  nmol/l (21) | 800 | PBO | 16 wks | - At 16 wks, significant (p<0.0001) increase in RBC folate with MMS (mean±SED, 1430±53 mmol/l) vs. PBO (583±33 mmol/l); steady state not reached with MMS at 16 wks - With MMS vs. PBO, significantly (p<0.0001) greater response rate (100% vs. 10%) and sustained treatment response (100% vs. 5%) for the target RBC folate level (>906 nmol/l) - Increase in RBC folate significantly (p<0.0001) greater in women with lower baseline RBC folate than higher levels - Target RBC folate level reached by 4.2±3.5 wks with MMS - At 16 wks, significant (p<0.0001) change from baseline in:   - Plasma folate with MMS (+292.3%) vs. PBO (-25.6%)   - Total HCY with MMS (-30.1%) vs. PBO (+10.1%)   - Vitamin B12 with MMS (+24.6%) vs. PBO (-10.5%)   - Vitamin B2 with MMS (+12.1%) vs. PBO (-1.6%)   - Vitamin B6 with MMS (+71.6%) vs. PBO (-0.4%) | - Preventive RBC folate concentration of >906 nmol/l can be reached within 4 wks of supplementation with daily intake of MMS containing 800 mg folic acid - To prevent fetal malformations in early pregnancy, supplementation of folic acid in higher amounts and in combination with other vitamins should be considered |
| [Schaefer *et al.* 2016](#_ENREF_27)  (Germany) | Double-blind, PBO-controlled RCT | Healthy women (20) | Healthy women (20) | 800 (folic acid 400 μg & MTHF 451 μg) | PBO | 16 wks | - Proportion of women achieving target erythrocyte RBC folate threshold (906 mol/l) significantly (p<0.0001) higher with MMS than PBO:   - 70.0% vs. 5.3% at week 4   - 100% vs. 5.3% at week 16 - In plasma at 4 & 16 wks:   - HCY decreased by 19% with MMS, but remained constant with PBO   - Vitamin B6 increased by 64% & 66% with MMS but remained constant with PBO   - Vitamin B12 remained constant with MMS, but decreased by 8% & 10% with PBO | - MMS (containing folic acid and MTHF at equimolar amounts) was effective at replenishing erythrocyte folate to levels considered protective of NTD by 4 wks in healthy women of child-bearing potential |
| [Pilz *et al.* 2017](#_ENREF_25)  (Germany) | Single-center, open RCT | Healthy women (101) | Healthy women (100) | 400 (containing 200 IU vitamin D3 | MMS 800 (containing 800 IU vitamin D3 | 8 wks | - Increase in 25(OH)D with both forms of MMS, although significantly (p<0.001) greater median increase with 800 IU than 200 IU vitamin D3 - Even the lower dose of vitamin D3 was sufficient to enable 70.4% of women to achieve adequate 25(OH)D levels (≥50 nmol/l) | - Daily MMS containing 200 IU vitamin D3 during wintertime in Germany was sufficient to achieve 25(OH)D level ≥50 nmol/l in the majority women of childbearing age |
| [Obeid *et al.* 2018](#_ENREF_18)  (Germany) | Single-center, open RCT | Healthy women (101) | Healthy women (100) | 400 | 800 | 8 wks | - 88% of women had RBC folate <906 nmol/l at baseline (associated with increased risk of NTD) - Significantly (p<0.001) higher RBC folate levels with folic acid 800 vs. 400 μg/day at 4 wks (928±330 vs. 805±363 nmol/L) and 8 wks (1218±435 vs. 1021±414 nmol/L) - Significantly more women achieved desirable RBC folate levels at 4 wks (45.5 vs. 31.3%; p=0.041) and 8 wks (83.8 vs. 54.5%; p<0.001) with 800 vs. 400 μg folic acid | - Women with low RBC folate unlikely to achieve desirable levels within 4-8 weeks, unless they received folic acid 800 μg/day   - Serum and RBC folate concentrations did increase with folic acid 400 μg/day for 4-8 wks, but most women still had RBC folate ≤906 nmol/l - Supplementation recommendations for folic acid 400 μg/day are insufficient in countries not applying fortification. |
| ***Outcomes preconception***  *Women trying to conceive* | | | | | | | | |
| [Dudás](#_ENREF_10) *[et al.](#_ENREF_10)* [1995](#_ENREF_10) | Subanalysis of double-blind, PBO-controlled RCT | Women trying to conceive (497) | Women trying to conceive (513) | 800 | ‘Trace-element’ PBO (Cu 1 mg, Mn 1 mg, Zn 7.5 mg, vit. C 7.5 mg) | At least 1 mo PC, but up to pregnancy or 12 mo PC | - More regular menstrual cycle with MMS vs. PBO, mainly in women with irregular cycles (a trend not seen with PBO) | - Even in healthy, well-nourished women, supplementation with folic acid and other micronutrients improve follicular development, mainly in women with irregular cycles |
| [Czeizel](#_ENREF_8) *[et al.](#_ENREF_8)* [1996](#_ENREF_8)  (Hungary) | Subanalysis of double-blind, PBO-controlled RCT | Women trying to conceive (3953) | Women trying to conceive (3952) | 800 | ‘Trace-element’ PBO (Cu 1 mg, Mn 1 mg, Zn 7.5 mg, vit. C 7.5 mg) | PC until end 1stT (max. 7 mo) | - Significantly (p=0.001) higher rate of conception with MMS (71.3) vs. PBO (67.9%); after exclusion of ‘unsupplemented’ subgroups, the difference remained significantly (p<0.05; OR 1.1 (95% CI 1.00-1.21)) in favor of MMS - Mean number of cycles required to conceive significantly lower with MMS (3.8±3.2) than PBO (4.0±3.3) - The use of fertility drugs was similar between the groups | - The slight but significantly higher rate of conception after MMS represents an increase in fertility of 5%, with fewer cycles required to achieve conception - The mechanism for increased fertility may be related to more regular female cycles because of improved hormonal status |
| [Arzhanova *et al.* 2009](#_ENREF_1)  (Russia) | Case series | Women with HHC and recurrent miscarriage (60) | — | 800 | — | 3 mo PC | With MMS:   - Blood HCY levels were significantly (p<0.05) reduced from (mean±SD) 12.8±1.5 to 7.8±0.95 μmol/l | - MMS led to standardization of HCY levels in women with recurrent miscarriage |
| [Wang *et al.* 2017](#_ENREF_32)  (China) | Single-center, RCT | Healthy, women planning pregnancy (21) | Healthy, women planning pregnancy (17) | 800 | Folic acid alone (400 μg) | 12 wks | - At 4 wks, significantly (p<0.01) more women achieved target erythrocyte folic acid concentration (906 nmol/l) with MMS (95.2%) than folic acid alone (58.8%) - With MMS vs. folic acid alone compared with baseline:   - Significant (p<0.05) increase in serum vitamin B12 vs. no significant change   - Significant (p<0.05) decrease in serum HCY levels from 2 wks vs. 8 wks | - MMS achieved erythrocyte folic acid threshold level for the prevention of NTDs more rapidly than folic acid alone, with concurrent faster reduction in HCY levels and significant increase in serum vitamin B12 levels |
| [Kuroda *et al.* 2021](#_ENREF_13)  (Japan) | Consecutive case series | Infertile women (205) | — | 800 ± vitamin D 25 or 50 μg | — | Until serum folate ≥7.0 ng/mL and HCY <13.5 nmol/mL achieved and 6 mo (pregnancy outcomes) | - In 49 women with serum folate level <7.0 ng/ml and/or HCY >13.5 nmol/ml, MMS significantly (p<0.0001):   - Increased folate levels (from 5.8±0.9 to 19.2±4.0 ng/ml)   - Decreased HCY levels (from 8.2±3.1 to 5.8±0.8 nmol/l)   - Similar increases in folate/decreases in HCY across MTHFR genotypes   - Serum folate levels increased to >7.0 ng/ml in all women with NTD risk, and no women had hyperhomocysteinemia after 1 mo - In 156 women with MTHFR genotypes, within 6 months:   - Cumulative pregnancy rates ranged from 39.2-57.7%   - Miscarriage rates ranged from 6.5-8.7%   - No significant difference between genotypes | - At baseline in infertile women, 26.3% had serum folate <7 ng/mL (a risk factor for NTD) and >95% had vitamin D insufficiency or deficiency - Regardless of MTHFR genotype, MMS + additional vitamin D improved folate and HCY levels, to minimize the risk of NTD after only 1 month of supplementation |
| [Radzinsky *et al.* 2021](#_ENREF_26)  (Russia) | Multicenter, observational intervention study | Women planning pregnancy (200) | — | 400 (MTHF-Ca) | — | Mean 101.3±2.3 days | - In 45% of participates, blood folate was <7 ng/ml in 45% of participants at - MMS increased plasma folate levels, from mean 5 ng/ml at baseline to 12.8 at day 84 to 13.6 at the final visit - The increase in plasma folate was achieved with all MTHFR gene variants - In overweight women, median plasma folate reached 11.4 ng/ml at day 84 and 12.6 ng/ml after 3 mo | - Correction of micronutrient imbalances is an important mechanism for reducing the incidence of malformations, maternal and infant mortality, intrauterine fetal death, and other serious complications |
| ***Outcomes preconception***  *Women undergoing fertility treatment including IVF* | | | | | | | | |
| [Özkaya & Nazıroğlu 2010](#_ENREF_21)  (Turkey) | PBO-controlled RCT and age-matched controls | Women undergoing IVF (26) | Paired FF & serum samples from women undergoing IVF (30) and age-matched healthy women (13) | 800 | PBO in all controls | 45 days PC | Compared to healthy and IVF controls, MMS in IVF women led to significant (p≤0.01):   - Decrease in FF and serum levels of lipid peroxidation - Increase in serum levels of GSH and vitamins C and E - Increase in FF levels of GSH-Px and vitamins C and A   In IVF controls vs. MMS IVF women and healthy controls, significant (p<0.05):   - Increase in FF and serum LP levels - Decrease in serum vitamins A and C levels, and FF vitamin C and GSH-Px levels | - MMS in serum and FF of women undergoing IVF may strengthen the antioxidant defence system by decreasing oxidative stress - MMS probably ameliorates antioxidant changes through its free radical scavenging |
| [Özkaya *et al.* 2011](#_ENREF_22)  (Turkey) | PBO-controlled RCT with some age-matched controls | Women undergoing IVF (26) | Paired FF & serum samples from women undergoing IVF (30) and age-matched healthy women (13) | 800 | PBO in all controls | 45 days PC | MMS vs. untreated IVF women led to:   - Significantly higher FF levels of Cu (p<0.01), Zn (p<0.05), Se (p<0.05), and Al (p<0.01), but significantly lower FF levels of Fe (p<0.001) - Significantly higher serum levels of Cu, Zn, Se, and Mg (p≤0.05)   In untreated IVF women vs. healthy controls:   - Significantly lower FF levels of Se (p<0.05) and Zn (p<0.01), but significantly higher FF levels of Fe and Al (p<0.05) - Significantly lower serum levels of Cu, Zn, and Se (p<0.05) | - Severe dietary deficiencies of trace elements, including Cu, Se and Zn, are commonly seen in IVF patients - MMS normalized levels of these trace elements in serum and FF of women undergoing IVF |
| [Sun *et al.* 2013a](#_ENREF_29)  (China) | PBO-controlled RCT | Women undergoing IVF (30) | Women undergoing IVF (30) and healthy women (25) | 800 | PBO | 60 days PC | - MMS vs. untreated IVF women led to:   - Significantly higher serum levels of Cu (p<0.05), Zn (p<0.01) and Mn (p<0.05)   - Significantly higher FF levels of Cu (p<0.01) and Zn (p<0.05), but significantly lower (p<0.001) Fe levels - In untreated IVF women vs. healthy controls, significantly lower (p<0.05) serum levels of zinc and copper | - MMS might normalize trace element levels in the serum and FF of women undergoing IVF - Trace elements could impact endocrine gland function, and target tissue activity and hormone bio-activity, thereby playing a major role in the reproductive process |
| [Luddi *et al.* 2016](#_ENREF_15)  (Italy) | Crossover trial | Older women undergoing IVF (18) | — | 800  (2^nd^ cycle) | No supplement (1^st^ cycle) | 3 mo pre-IVF cycle | - Significant (p<0.05) improvement in total antioxidant capacity in FF and serum with MMS vs. no supplement - Significant (p<0.05) protection from oxidative damage in FF and serum proteins with MMS vs. no supplement - Significantly (p=0.01) fewer poor quality oocytes retrieved with MMS (1.20±0.77) vs. no supplement (1.88±1.01) - Pregnancy rate 17.7% with MMS | - MMS begun 3 mo before IVF cycle protected the follicular microenvironment from oxidative stress, thus improving retrieval of good quality oocytes |
| [Ogawa *et al.* 2023](#_ENREF_19)  (Japan) | Hospital-based, retrospective analysis | Infertile women with history of IVF/ICSI failure (1060) | | | | | - Significant (p<0.0001) relationship between higher HCY levels and lower fertilization rate - Significant (p<0.0001) inverse relationship between HCY levels and serum 25(OH)D - Trend towards lower AMH in women with higher HCY levels (p=0.05) | - In patients with a history of IVF/ICSI failure, higher HCY levels associated with lower 25(OH)D levels and lower fertilization rates |
|  | Prospective interventional study | Infertile women with history of FET failures (26) | Infertile women with history of FET failures (30) | 800 | No supplement | 12 wks (PC) | - At baseline, women receiving MMS had significantly (p=0.01) lower 25(OH)D levels and higher HCY levels than controls - After 12 wks of MMS:   - Significant (p=0.001) decrease in HCY levels vs. baseline | - In women with history of FET failure, MMS reduced HCY levels - MMS and HCY level monitoring may constitute a novel intervention for improving IVF/ICSI pregnancy outcomes |
| ***Outcomes first/second trimester***  *Pregnant women* | | | | | | | | |
| [Czeizel *et al.* 1992](#_ENREF_7)  (Hungary) | First 1000 pregnancies from double-blind, PBO-controlled RCT | Pregnant women (500) | Pregnant women (500) | 800 | ‘Trace-element’ PBO (Cu 1 mg, Mn 1 mg, Zn 7.5 mg, vit. C 7.5 mg) | PC until end 1stT (max. 7 mo) | - Significantly (p<0.01) lower rate of vertigo, nausea, and vomiting with MMS vs. PBO in early pregnancy (0.6% vs. 2.4%, respectively) and end of 1stT (3.4% vs. 7.4%) - Similar amount of weight gain in each group from preconception to end of 1stT | - The significantly lower rate of vertigo, nausea, and vomiting with MMS is likely due to a combined effect of micronutrients that optimize nutritional status and metabolism |
| [Czeizel 1994](#_ENREF_4)  (Hungary) | Final pooled analysis of double-blind, PBO-controlled RCT | Informative offspring (2471) | Informative offspring (2391) | 800 | ‘Trace-element’ PBO (Cu 1 mg, Mn 1 mg, Zn 7.5 mg, vit. C 7.5 mg) | PC until end 1stT (max. 7 mo) | - Significant (p=0.01) difference in number of NTD with MMS (no cases) vs. PBO (six cases) - Significantly (p=0.018) lower total rate of CA with MMS (5.91%) than PBO (7.61%); after exclusion of six NTD, difference remained significant (p=0.04; RR 0.80 (95% CI 0.65-0.99)) | - Protective CA effect with MMS may be related to folic acid and other vitamin intakes - Reduction in CA mainly explained by lower rates of congenital cardiovascular malformations, urinary system defects, congenital hypertrophic pyloric stenosis |
| [Czeizel *et al.* 2004](#_ENREF_6)  (Hungary) | Two-cohort trial (TCT) using patients from double-blind, PBO-controlled RCT recruited at 14 wks gestation | Women with informative offspring (3056) | Matched controls (3056) | 800 | No supple­ment | PC until end 1stT (max. 7 mo) | - Significantly fewer cardiovascular CAs with MMS (31) than controls (50) (OR 0.60 (95% CI 0.38-0.96)) - Mainly due to fewer ventricular septal defects with MMS (5) than controls (19) (OR 0.26 (95% CI 0.09-0.72)) - Significantly fewer stenosis/atresia of PUJ with MME (2) than in controls (13) (OR 0.19 (95% CI 0.04-0.86)) - Fewer NTD with MMS (1) than in controls (9) (OR 0.11 (95% CI 0.01-0.91)) (note: 41/3056 MMS had family history of NTD) | - Protective effect of MMS against NTD confirmed - MMS leads to primary prevention of some major structural defects and is of great public importance - Note: high-risk women could not ethically be excluded from receiving MMS, so non-supplemented women had predominantly no or little morbidity and no history of adverse pregnancy outcomes—MMS still provided significant medical benefit |
| [Czeizel 2004](#_ENREF_5)  (Hungary) | Pooled analysis of the above RCT & TCT, plus results from the HCCSCA | RCT + TCT: offspring (5527)  HCCSCA: malformed offspring (22843) | RCT + TCT: offspring (5447)  HCCSCA: controls without CA (38151) | RCT + TCT: 800  HCCSCA: folic acid only (300-900 or NR) | RCT + TCT: no supplement HCCSCA: folic acid only (300-900 or NR) | PC & 1stT | RCT + TT: MMS incl. folic acid reduced risk of:   - NTD vs. no supplement (OR 0.08 (95% CI 0.01-0.47)) - Obstructive urinary CA (OR 0.19 (95% CI 0.04-0.86)) - Cardiovascular CA (OR 0.57 (95% CI 0.39-0.85)) - Congenital pyloric stenosis (OR 0.20 (95% CI 0.04-0.90))   HCCSCA:   - Folic acid only prevented:   - NTD (1^st^ mo: OR 0.68 (95% CI 0.47-0.97))   - Posterior cleft palate (1^st^ mo, OR 0.50 (95% CI 0.28-0.89))   - Cardiovascular CA (1^st^ mo: OR 0.81 (95% CI 0.68-0.96); 2^nd^ mo, OR 0.75 (95% CI 0.65-0.86))   - Rectal/anal atresia/stenosis (2^nd^ mo: OR 0.39 (95% CI 0.17-0.88))   - New candidate CA (hypospadias, poly/syndactyly, multiple CA), esp. when taken during 1^st^ mo - When the pooled analysis and HCCSCA were compared, MMS was more effective than folic acid alone at reducing the risk of NTD and CA | - Intervention trials: 92% of NTD may be prevented using MMS containing physiological dose of folic acid (800 μg) during the periconceptional period, and can prevent certain CA - HCCSCA: folic acid supplementation alone also reduced NTD and the overall occurrence of CA - However, MMS was more effective than folic acid alone - Daily use of MMS including 400-800 μg can be recommended with healthy diet and lifestyle in women who want to do their best to prevent NTD and some CA |
| [Pasman *et al.* 2005](#_ENREF_24)  (Russia) | Observational and retrospective analysis of case series | Pregnant women with HA (obs.) (28) &  healthy pregnant women (13) | Pregnant women with HA (retro.) (23) &  healthy pregnant women (11) | 800 (60 mg iron) | No supplement | 3-6 mo PC and through-out gestation | - In women with HA, lower rate of:   - Threatened miscarriage with MMS (35.8% 1stT; 25% 2ndT; 21.4% 3rdT) vs. no supplement (86.8% 1stT & 2ndT; 56.4% 3rdT)   - Placental insufficiency with MMS vs. no supplement (p<0.005)   - Functional CA requiring correction with MMS (17.9%) vs. no supplement (36.0%)   - No CA with MMS (one case with no supplement) - In healthy women, lower rate of placental insufficiency with MMS (15.4%) vs. no supplement (36.8%) - In women with HA and healthy women, significantly lower rate of IDA with MMS vs. no supplement | - Pregnant women with hyperandrogenic conditions belong to a high-risk group regarding premature deliveries and placental insufficiency—the risk of both were reduced with MMS vs. no supplement - MMS also reduced the risk of IDA |
| [Mozgovaya *et al.* 2011](#_ENREF_17)  (Russia) | Case series | Pregnant, hospitalized women (60) (mostly for threatened miscarriage, 78%) | — | 800 (with 60 mg iron) | — | 1stT and 2ndT | With MMS:   - No decrease in hemoglobin by 2ndT - Significant increase in Fe levels to physiological norm - Levels of Fe, ferritin, and transferrin transport proteins did not change significantly - No increase in coagulative potential in the majority of women - Significant (p<0.05), 2-fold decrease in HCY (from 9.4±2.1 to 4.6±0.8 μmol/l) - Normalization of total antioxidant activity and increase in total anti-radical activity in 2ndT (p<0.05) - Mild gestosis in women with multiple gestation (5.5%), but no premature or operative delivery; frequency lower than in general population | - MMS reduces the risk of pregnancy complications, maintains wellbeing of the future mother, and the proper development of the fetus - MMS has an effective preventive role in IDA - MMS has a preventive, but also therapeutic effect in terms of correction of hyperhomocysteinemia |
| [Sun *et al.* 2013b](#_ENREF_30)  (China) | Retrospective analysis | Women pregnant after IVF (2693) | Women pregnant after IVF (1502) | 800 | No supplement | 3 mo from day of transplant | - No NTD with MMS vs. six cases with no supplement (i.e. four cases per thousand pregnancies) (p=0.002) | - Taking MMS after IVF can prevent fetal NTD |
| [Vanderlelie *et al.* 2016](#_ENREF_31)  (Australia & NZ) | Prospective, longitudinal birth cohort  study | Pregnant women, stratified by weight (719) | Pregnant women, stratified by weight (1542) | 800 (identified by 52% of MMS users) | No supplement (n=1066)  or  folic acid only (likely 800 μg; n=476) | 1stT | - Overall occurrence of pre-eclampsia significantly lower with MMS (0.97%) and folic acid only (1.26%) vs. no supplement (2.9%) - After adjustment, risk of pre-eclampsia:   - 66% lower with MMS than no-supplement in all pregnant women (adjusted OR 0.33 (95% CI 0.14-0.75))   - 52% lower with MMS in overweight/obese women (adjusted OR 0.48 (95% CI 0.27-0.86)   - Protective effect of MMS increased with increasing BMI, from 55% reduction in overweight women (adjusted OR 0.45 (95% CI: 0.30, 0.86)) to 62% reduction in obese women (adjusted OR 0.38 (95% CI: 0.16, 0.92))   - Reduction also seen with MMS in lean women, but did not reach 5% significance level (adjusted OR 0.60 (95% CI 0.39-1.36)) | - First trimester multivitamin/mineral supplementation is beneficial in reducing the risk of pre-eclampsia, particularly in women who are overweight or obese - Improvements in baseline micronutrient status (particularly vitamins A, B1 & D, folate, Ca, Fe, Mg, Zn—reported as reduced in Australian population) may have contributed to beneficial effect of MMS |
| [Lin](#_ENREF_14) *[et al.](#_ENREF_14)* [2020](#_ENREF_14)  (China) | Retrospective analysis | Pregnant women (5301)  MMS in early pregnancy (3929) | Pregnant women (2985)  Folic acid only in early pregnancy (4357) | 800 (60 mg iron) | Folic acid only (400 μg) | 1stT to 3^rd^T | - Using MMS vs. folic acid alone during pregnancy:   - Significantly (p<0.01) reduced the incidence of IDA (1.2% vs. 25.3%)   - Reduced the incidence of pre-eclampsia (0.5% vs. 1.2%), fetal macrosomia (3.8% vs. 4.8%), and postpartum hemorrhage (3.2% vs. 7.9%) - Using MMS vs. folic acid alone during early pregnancy:   - Significantly reduced the incidence of IDA (1.3% vs. 17.6%)   - Reduced pre-eclampsia (0.3% vs. 1.1%), intrahepatic cholestasis of pregnancy (3.4% vs. 5.3%), premature birth (3.7% vs. 5.4%), postpartum hemorrhage (3.8% vs. 5.8%), and low birth weight (2.7% vs. 3.7%) | - Using MMS in pregnancy can reduce the risk of adverse pregnancy outcomes, particularly IDA. Using MMS is especially important during early pregnancy. |
| [Ou & Yu 2020](#_ENREF_20)  (China) | Retrospective analysis | Women with unexplained recurrent miscarriage (106) | Women with unexplained recurrent miscarriage (65) | MMS (800) + ASA (100 mg for 3 mo PC then 75 mg) + prednisone (5 mg) | Folic acid alone (400 μg) | 3 mo PC until end of 1stT | - Rate of successful pregnancy (number of pregnancies divided by total number of women) was similar between MMS (89.6%) and control groups (92.3%) - Rate of successful treatment (number of women with successful treatment *^b^* divided by number of women with successful pregnancy) significantly (p<0.001) higher in MMS (86.3%) than control group (53.3%) - MMS therapy associated with higher odds of successful treatment (number of spontaneous pregnancy losses associated with lower odds) | - MMS + ASA + prednisone therapy is a good treatment option for women with unexplained recurrent miscarriage |
| [Ogawa *et al.* 2023](#_ENREF_19)  (Japan) | Prospective interventional study | Infertile women with history of FET failures (26) | Infertile women with history of FET failures (30) | 800 | No supplement | 12 wks (PC) | - After 12 wks of MMS:   - Significant (p<0.05) reduction in miscarriage vs. control (95% CI 0.02-1.20)   - No significant (p=0.97) improvement in clinical pregnancy rates vs. control (95% CI 0.15-1.25) | - In women with history of FET failure, MMS improved pregnancy outcomes - MMS and HCY level monitoring may constitute a novel intervention for improving IVF/ICSI pregnancy outcomes |
| ***Outcomes second and third trimester*** | | | | | | | | |
| [Khodova & Murashko 2006](#_ENREF_11)  (Russia) | Case series | Pregnant women with anemia, gestosis (i.e. pre-eclampsia) and thyroid disorders (87) | — | 800 (with 60 mg iron) | — | 2ndT & 3rdT (mean 47.3±2.6 days) | - At baseline in anemic women (n=33):   - Folic acid levels were below physiological levels   - Hemoglobin, erythrocyte and hematocrit levels were low - By the end of pregnancy in anemic women, MMS led to significant increases in:   - Folic acid levels to physiological standards (p<0.0001)   - Mg (p<0.05), Fe (p<0.0001)   - Hematological values: hemoglobin (p<0.01), erythrocytes (p<0.001), hematocrit (p<0.001), serum Fe (p<0.05) - By the end of pregnancy in women with pre-eclampsia (n=35), MMS led to significant increases in:   - Folic acid (p<0.05), P (p<0.05), Mg (p<0.0001), Ca (p<0.05), Fe (p<0.0001)   - Hematological values: hemoglobin (p<0.0001), erythrocytes (p=0.02), hematocrit (p<0.005), serum Fe (p<0.005) - By the end of pregnancy in women with thyroid disorders (n=19), MMS led to significant increases in:   - Folic acid (p<0.0001), P (p<0.02), Fe (p<0.0001)   - Note: MMS did not contain iodine | - MMS is effective for the prevention and combination treatment of mild and moderate anemia and gestosis, and enables individual and appropriate hormonal correlation with iodine drugs in women with thyroid disorders - Addressing deficiencies in folic acid, as well as Fe, Mg and other trace substances, has a beneficial effect on the course of pregnancy, decreasing the rate of obstetric complications |
| [Arzhanova *et al.* 2009](#_ENREF_1)  (Russia) | Case series | Women with HHC and recurrent miscarriage (60) | — | 800 | — | 3 mo PC and during the entire preg­nancy | With MMS:   - Term births increased by 4 times - Preterm delivery decreased by 6.6 times (5.8±3.2%) vs. previous pregnancies (40.0±11.0%) - Reduction in incidence of spontaneous miscarriages (6 times) and non-developing pregnancies (10 times) | - MMS led to reductions in spontaneous miscarriages and preterm deliveries in women with recurrent miscarriage |
| [Kurmacheva *et al.* 2018](#_ENREF_12)  (Russia) | Retrospective comparative analysis | Mothers (147) | Mothers (229) | 800 + potassium iodide (250 μg) | No supplement/not regular users | 3rdT and post-partum | In women not regularly using MMS/no MMS:   - Multiple hypovitaminosis was common in pregnant women (72.6% of mothers)   - Associated with obstetric and perinatal complications: anemia, chronic fetal hypoxia, acute infectious diseases during pregnancy, pathological delivery in mothers, adaptation disorders, perinatal damage in the CNS, acute infectious diseases in neonates (RR 1.55-5.48; *p*<0.05) - Low concentrations of vitamins A, B1, B2, C, beta-carotenoids, iodine, Fe, Zn and Se in breast milk of lactating women   Regular use of MMS from early stages of pregnancy (+ additional potassium iodide) substantially:   - Increased blood Fe, Cu, Zn, Se, and vitamins A, E, C, B1, B2 in 3rdT (p≤0.0002) - Reduced rate of pre-eclampsia, anemia, chronic fetal hypoxia, acute infectious diseases, and active labor abnormalities in mothers - Reduced disharmonic physical development, adaptation disorders, perinatal damage in the CNS, and acute infectious diseases in neonates (by a factor of 1.3−3; *p*<0.05) | - Regulation of micronutrient intake during pregnancy resulted in an improvement in obstetric and perinatal outcomes |
| [Massari](#_ENREF_16) *[et al.](#_ENREF_16)* [2020](#_ENREF_16)  (Italy) | Open RCT | Pregnant women (65) | Pregnant women (76) | 400 (226 in the form of MTHF-Ca) + DHA (200 mg) | No supplement | GW 13-15 until delivery | In pregnant women with MMS vs. no supplement:   - Significantly (p<0.0001) greater increase in RBC DHA levels: estimated treatment difference 0.96 (95% CI 0.61-1.31)   - Greater increases in women at lower ranges with MMS, who reached RBC DHA threshold (5%) by study end - Significantly better:   - RBC DHA/TFA ratio: estimated difference 0.01 (95% CI 0.006-0.013) (p<0.0001)   - Omega-3 index: estimated difference 1.00 (95% CI 0.64-1.37) (p<0.0001)   - 25(OH)D: estimated difference 3.96 (95% CI 0.88-7.04) μg/l (p=0.0122)   In infants with maternal MMS vs. no supplement, significantly:   - Thicker skinfold thickness (p=0.0292) - Greater bone density (p=0.0486) | - MMS + DHA in pregnant women in an industrialized setting can:   - Complement dietary intake   - Significantly improve maternal DHA and vitamin D status (important finding considering the essential roles of DHA and vitamin D) |
| ***Outcomes post-partum*** | | | | | | | | |
| [Paoletti *et al.* 2013](#_ENREF_23)  (Italy) | RCT | Healthy women post-partum without risk factors for depression (424) | Healthy women post-partum without risk factors for depression (428) | 800 | Ca (500 mg) + vitamin D3 (400 IU) | 3-30 days after delivery | - Significant (p<0.05) and comparable increase in hematological parameters in both groups, including blood iron, hemoglobin and ferritin - Significantly (p<0.05) greater improvement in postnatal depression (i.e. decrease in EPDS score) with MMS vs. control   - Particularly evident in women with basal EPDS <12 | - MMS favorably modulates brain functions antagonizing the evolution to post-partum depression |
| [Schaefer *et al.* 2020](#_ENREF_28)  (Germany) | Double-blind PBO-controlled RCT | Healthy lactating women from high-income country (35) | Healthy lactating women from high-income country (35) | 500 + DHA (200 mg) + lutein (250 μg) | PBO (no active ingredient, apart from iodine 225 mg) | From 4-6 wks after delivery for 12 wks | - In maternal milk:   - DHA increased by 30% with MMS but decreased with PBO (LS mean difference 0.15 (0.11-0.19); p<0.0001)   - EPA increased with MMS but decreased with PBO (LS mean difference 0.0110 (0.0006-0.0214); p=0.038)   - Beta-carotene increased with MMS but decreased with PBO (LS mean difference 28.4 (15.0-41.9); p<0.0001) - In maternal blood:   - DHA increased by 17% with MMS but decreased with PBO (LS mean difference 15.66 (11.96-19.36); p<0.0001)   - EPA increased by 4% with MMS but decreased with PBO (LS mean difference 2.21 (0.44-3.98); p=0.0155)   - 25(OH)D increased by 24% with MMS but decreased with PBO (LS mean difference 7.82 (4.36-11.28); p<0.0001)   - Folic acid increased with MMS but decreased with PBO (LS mean difference 21.20 (17.84-24.56); p<0.0001)   - Vitamin B12 increased by 12% with MMS but decreased with PBO (LS mean difference 89.89 (31.45-148.3); p=0.0031)   - Lutein increased by 4% with MMS, but decreased with PBO (LS mean difference 21.13 (4.15-38.31); p=0.0157)   - Beta carotene increased by 49% with MMS but decreased with PBO (LS mean difference 296.35 (183.06-409.64); p<0.0001)   - HCY decreased with MMS but increased with PBO (LS mean difference -1.63 (-2.27 to -0.99); p<0.0001)   - Docosatetraenoic acid decreased with MMS and PBO, but significantly more with MMS (LS mean difference -0.46 (-0.86 to -0.05); p=0.0270) | - In lactating women with no overt nutritional deficiencies, macro- and micronutrient intake from food alone was often insufficient - Maternal MMS + DHA + lutein during the lactation period led to:   - Significant increases in maternal milk levels of DHA, EPA and beta-carotene   - Significant increases in maternal blood levels of DHA, EPA, 25(OH)D, folate, vitamin B12, lutein, and beta-carotene.   - Significant decreases in maternal blood levels of HCY and docosatetraenoic acid |

*^a^* Elevit™ (Bayer); it should be noted that slight variations in micronutrient content were used in these studies, as outlined in Table 1 of the article. *^b^* Defined as a 12-week pregnancy with an obvious embryo and embryonic heart revealed by ultrasound examination, nuchal translucency thickness <0.25 cm, size consistent with gestational age, and no early malformations. Abbreviations: 1stT, first trimester; 25(OH)D, 25-hydroxyvitamin D3; 2ndT, second trimester; 3rdT, third trimester; AMH, anti-mullerian hormone (plays a role in the development of reproductive organs); ART, assisted reproductive technology; ASA, acetylsalicylic acid; BMI, body mass index (kg/m^2^); CA, congenital abnormalities; CI, confidence interval; CNS, central nervous system; DHA, docosahexaenoic acid; EPA, eicosapentaenoic acid; EPDS, Edinburgh Depression Postnatal scale; FET, freeze embryo transfer; FF, follicular fluid; GSH, reduced glutathione; GSH-Px, glutathione peroxidase; GW, gestational week; HA, hyperandrogenism; HCCSCA, Hungarian Case-Control Surveillance of Congenital Abnormalities; HCY, homocysteine; HHC, hyperhomocysteinemia; ICSI, intracytoplasmic sperm injection; IDA, iron-deficiency anemia; IQR, interquartile range; IVF, in vitro fertilization; LS, least squares; MMS, multiple micronutrient supplementation; mo, months; MTHF(-Ca), L-5-methyltetrahydrofolate (calcium salt) (NB. 225 μg L-5-MTHF-Ca corresponds to 200 μg folic acid); MTHFR, methylenetetrahydrofolate reductase; NR, not reported; NTD, neural tube defects; OR, odds ration; PBO, placebo; PC, preconception; PUJ, pelvic ureteric junction; RBC, red blood cell; RCT, randomized, controlled trial; RR, relative risk; SD; standard deviation; SEM, standard error of the mean; TFA, total fatty acids; wks, weeks.

**Supplementary Table 2.** Safety and tolerability of supplementation with multiple micronutrients *^a^* throughout preconception, pregnancy and lactation.

| **Study (country)** | **Study design** | **Study group (N)** | **MMS:  folic acid (μg)** | **Duration** | **Adverse events reported within the trial for MMS** |
| --- | --- | --- | --- | --- | --- |
| ***Healthy women*** | | | | | |
| [Schaefer *et al.* 2016](#_ENREF_27)  (Germany) | Double-blind, PBO-controlled RCT | Healthy women (20) | 800 (folic acid 400 μg & MTHF 451 μg) | 16 wks | - 129 TEAEs, of which 12 (9.3%) were considered treatment-related   - MMS: 87 TEAEs in 15 (75.0%) subjects - Most common AEs overall were gastrointestinal (MMS 55% vs. PBO 15%), such as diarrhea (20% vs. 0%), flatulence (20% vs. 0%), and nausea (30% vs. 5%), which were more often considered treatment-related - No serious or severe, treatment-related AEs, none led to discontinuation, and all had resolved at the end of the study |
| [Pilz *et al.* 2017](#_ENREF_25)  (Germany) | Single-center, open RCT | Healthy women (201) | MMS  (800 μg folic acid + 800 IU vitamin D3 or 400 μg folic acid + 200 IU vitamin D3) | 8 wks | - Of 501 reported AEs, only 10 were classified as intolerance reactions to MMS - No serious AEs (including deaths) were reported |
| [Radzinsky *et al.* 2021](#_ENREF_26)  (Russia) | Multicenter, observational intervention study | Women planning pregnancy (200) | 400 (MTHF) | Mean 101.3±2.3 days PC | - No adverse events recorded in any patient - No deviations in clinical and biochemical blood tests found |
| ***Pregnant women*** | | | | | |
| [Arzhanova *et al.* 2009](#_ENREF_1)  (Russia) | Case series | Women with HHC and recurrent miscarriage (60) | 800 | 3 mo PC and during preg­nancy | - No adverse events observed during the study |
| [Mozgovaya *et al.* 2011](#_ENREF_17)  (Russia) | Case series | 60 women hospitalized for threatened miscarriage | 800 | 1stT and 2ndT | - No changes in blood biochemical parameters or increase in coagulative potential   - Increase in fibrinogen level >4.0 g/l in seven patients (11.7%) by 2ndT, requiring preventive endothelial protector therapy - Side effects reported in 2 out of 60 pregnant women (3.3%):   - One pregnant woman from 3rd day of taking MMS experienced gastrointestinal disorder (diarrhea), the other woman had exacerbation of chronic neurodermatitis |
| [Massari *et al.* 2020](#_ENREF_16)  (Italy) | Open RCT | Pregnant women (65) | 400 (200 folic acid + 226 MTHF-Ca) + DHA (200 mg) | Gestational week 13-15 until delivery | - Safety outcomes comparable between groups:   - 125 (71.0%) women reported at least one pertinent TEAE (232 TEAEs overall); 23 (13.1%) reported them as serious (comparable number in each group)   - At least one TEAE pertinent to the fetus/child reported in 10 (5.7%) women (13 TEAEs overall); five (2.8%) reported them as serious. - In the maternal MMS group:   - 19 (21.8%) had one TEAE that led to permanent treatment discontinuation   - Three (3.5%) had at least one suspected related TEAE (vomiting, mild severity)   - One (1.6%) subject had one TEAE pertinent to the fetus/child that led to permanent discontinuation   - One fatality, unrelated to study treatment - No relevant changes in clinical laboratory parameters (i.e., hematology, kidney function, liver function, blood coagulation, C-reactive protein), apart from a decrease in mean ferritin levels in both groups during the study - Normal physical and gynecological examinations throughout |
| ***Long-term safety: infants*** | | | | | |
| [Czeizel & Dobó 1994](#_ENREF_3)  (Hungary) | Data from double-blind, PBO-controlled RCT (HOFPP) | Liveborn infants (mean 11 (range 8-21) mo old) after maternal MMS (1809) | 800  (‘Trace-element’ PBO used: Cu 1 mg, Mn 1 mg, Zn 7.5 mg, vit. C 7.5 mg; n=1872) | Maternal MMS PC until end 1stT (max. 7 mo) | - (Data from full and partial MMS intake combined as no significant differences between groups) - No significant differences between MMS vs. PBO, respectively, in:   - Mortality (9.6/1000 vs. 7.1/1000) (p=0.40)   - Rates of serious or chronic disorders overall (p=0.34)   - Somatic development: body weight (p=0.66), body length (p=0.08), head circumference (p=0.21)   - Mental (p=0.65) and behavioral development (p=0.49) and social skill quotient (p=0.76) - Significant differences between MMS vs. PBO, respectively, for:   - Atopic dermatitis, higher with MMS (15 vs. four cases; p=0.012); however, four of the 15 MMS infants had a parent with atopic dermatitis   - Asthma and wheezy bronchitis, higher with MMS (26 vs. eight cases) (p=0.0022); of these, six MMS infants had positive family history but no PBO infants did |
| [Dobó & Czeizel 1998](#_ENREF_9)  (Hungary) | Long-term (2 & 6 years) follow-up of HOFPP | Children after maternal MMS (323) | 800  (‘Trace-element’ PBO used: Cu 1 mg, Mn 1 mg, Zn 7.5 mg, vit. C 7.5 mg; n=302) | Maternal MMS PC until end 1stT (max. 7 mo) | - No adverse effects of periconceptional MMS on the long-term postnatal somatic and mental development of children - No significant differences between MMS vs. PBO, respectively, in:   - The rate of allergies overall (p=0.08 at 2 y; p=0.56 at 6 y), including atopic dermatitis (p=0.11 at 2 y; p=0.90 at 6 y)   - Anthropometric data, ophthalmological and audiological examinations, developmental variables, IQ or DQ at 2 y or 6 y - Significant difference between MMS vs. PBO, respectively, for:   - Otitis media, higher with MMS at 2 y (p=0.05) and 6 y (p=0.04) |
| ***Lactating women*** | | | | | |
| [Schaefer](#_ENREF_28) *[et al.](#_ENREF_28)* [2020](#_ENREF_28)  (Germany) | Double-blind PBO-controlled RCT | Healthy lactating women from high-income country (35) | 500 + DHA (200 mg) + lutein (250 μg) | From 4-6 wks after delivery for 12 wks | - No significant difference in the number of AEs observed between MMS and PBO - One mother each in the MMS and PBO groups experienced a treatment-related AE, considered to be:   - Moderate in the MMS group (peripartum hemorrhage) - No treatment-related AEs in infants reported with MMS or PBO - No women discontinued treatment and no mothers or infants died |

*^a^* Elevit™ (Bayer); it should be noted that slight variations in micronutrient content were used in these studies, as outlined in Table 1 of the article. Abbreviations: 1stT, first trimester; 2ndT, second trimester; AE, adverse event; DQ, development quotient; HOFPP, Hungarian Optimal Family Planning Programme; IQ, intelligence quotient; MMS, multiple micronutrient supplementation; MTHF(-Ca), L-5-methyltetrahydrofolate (calcium salt) (NB. 225 μg L-5-MTHF-Ca corresponds to 200 μg folic acid); PBO, placebo; PC, preconception; TEAE, treatment-emergent adverse event; wks, weeks; y, years.

# **References**

Arzhanova, O.N., Alyabyeva, E.A. & Shlyahtenko, T.N. (2009) 'Hyperhomocysteinemia in women with recurrent miscarriage', *Gynecol J*, 5: 53-55.

Brämswig, S., Prinz-Langenohl, R., Lamers, Y.*, et al.* (2009) 'Supplementation with a multivitamin containing 800 microg of folic acid shortens the time to reach the preventive red blood cell folate concentration in healthy women', *Int J Vitam Nutr Res*, 79: 61-70.

Czeizel, A E & Dobó, M (1994) 'Postnatal somatic and mental development after periconceptional multivitamin supplementation', *Arch Dis Childhood*, 70: 229-233.

Czeizel, A.E. (1994) 'The final data base of congenital abnormalities in the Hungarian randomised controlled trial of periconceptional multivitamin supplementation', *Acta Paediatr Hung*, 34: 19-44.

Czeizel, A.E. (2004) 'The primary prevention of birth defects: Multivitamins or folic acid?', *Int J Med Sci*, 1: 50-61.

Czeizel, A.E. , Dobó, M. & Vargha, P. (2004) 'Hungarian cohort-controlled trial of periconceptional multivitamin supplementation shows a reduction in certain congenital abnormalities', *Birth Defects Res A Clin Mol Teratol*, 70: 853-861.

Czeizel, A.E. , Dudas, I., Fritz, G.*, et al.* (1992) 'The effect of periconceptional multivitamin-mineral supplementation on vertigo, nausea and vomiting in the first trimester of pregnancy', *Arch Gynecol Obstet*, 251: 181-185.

Czeizel, A.E., Métneki, J. & Dudás, I. (1996) 'The effect of preconceptional multivitamin supplementation on fertility', *Int J Vitam Nutr Res*, 66: 55-58.

Dobó, M. & Czeizel, A. E. (1998) 'Long-term somatic and mental development of children after periconceptional multivitamin supplementation', *Eur J Pediatr*, 157: 719-723.

Dudás, I., Rockenbauer, M. & Czeizel, A. E. (1995) 'The effect of preconceptional multivitamin supplementation on the menstrual cycle', *Arch Gynecol Obstet*, 256: 115-123.

Khodova, S.I. & Murashko, L.E. (2006) 'Correction of deficiency of vitamins, minerals and trace substances during pregnancy. Influence of the multivitamin supplement Elevit on the blood composition of trace substances in pregnant women with anaemia, gestosis and thyroid disorders', *Consilium Medicum*, 6: 20-23.

Kurmacheva, N.A., Rogozhina, I.E., Chernenkov, Y.V. & Panina, O.S. (2018) 'Efficacy of Elevit® Vitamin-Mineral Complex Pronatal from early pregnancy for improvement of obstetric and perinatal outcomes', *Gynecology*, 20: 19-25.

Kuroda, K., Horikawa, T., Gekka, Y.*, et al.* (2021) 'Effects of Periconceptional Multivitamin Supplementation on Folate and Homocysteine Levels Depending on Genetic Variants of Methyltetrahydrofolate Reductase in Infertile Japanese Women', *Nutrients*, 13.

Lin, S., Xinghui, L., Peng, C. & al., et (2020) 'Retrospective cohort study of supplemental multivitamins during pregnancy and pregnancy outcome', *Chinese J Pract Gynaecol Obstet*, 36: 177-181.

Luddi, A. , Capaldo, A. , Focarelli, R. *, et al.* (2016) 'Antioxidants reduce oxidative stress in follicular fluid of aged women undergoing IVF', *Reprod Biol Endocrinol*, 14: 57.

Massari, M., Novielli, C., Mandò, C.*, et al.* (2020) 'Multiple micronutrients and docosahexaenoic acid supplementation during pregnancy: a randomized controlled study', *Nutrients*, 12.

Mozgovaya, EV, Prokopenko, VM, Oparina, TI & Novikova, TD (2011) 'Assessment of clinical efficacy of multivitamin and mineral complex Elevit Pronatal in prevention of iron deficiency anaemia and gestosis in pregnant women', *Obstet Gynecol (Moscow)*, 4: 89-94.

Obeid, R., Schön, C., Wilhelm, M.*, et al.* (2018) 'Response of Red Blood Cell Folate to Supplementation in Nonpregnant Women is Predictable: A Proposal for Personalized Supplementation', *Mol Nutr Food Res*, 62.

Ogawa, S., Ota, K., Takahashi, T. & Yoshida, H. (2023) 'Impact of Homocysteine as a Preconceptional Screening Factor for In Vitro Fertilization and Prevention of Miscarriage with Folic Acid Supplementation Following Frozen-Thawed Embryo Transfer: A Hospital-Based Retrospective Cohort Study', *Nutrients*, 15.

Ou, H. & Yu, Q. (2020) 'Efficacy of aspirin, prednisone, and multivitamin triple therapy in treating unexplained recurrent spontaneous abortion: A cohort study', *Int J Gynaecol Obstet*, 148: 21-26.

Özkaya, M.O. & Nazıroğlu, M. (2010) 'Multivitamin and mineral supplementation modulates oxidative stress and antioxidant vitamin levels in serum and follicular fluid of women undergoing in vitro fertilization', *Fertil Steril*, 94: 2465-2466.

Özkaya, M.O., Nazıroğlu, M., Barak, C. & Berkkanoglu, M. (2011) 'Effects of multivitamin/mineral supplementation on trace element levels in serum and follicular fluid of women undergoing in vitro fertilization (IVF)', *Biol Trace Elem Res*, 139: 1-9.

Paoletti, A.M. , Orrù, M.M. , Marotto, M.F. *, et al.* (2013) 'Observational study on the efficacy of the supplementation with a preparation with several minerals and vitamins in improving mood and behaviour of healthy puerperal women', *Gynecol Endocrinol*, 29: 779-783.

Pasman, N.M., Dudareva, A.V., Demina, S.G., Seemenova, N.V. & Bukhanovskaya, I.R. (2005) 'Experience in use of a multivitamin complex “Elevit Pronatal” in preconceptional period and during pregnancy for prevention of gestational complications in women with hyperandrogenic conditions', *Gynecol J*, 1.

Pilz, S., Hahn, A., Schön, C., Wilhelm, M. & Obeid, R. (2017) 'Effect of two different multimicronutrient supplements on vitamin D status in women of childbearing age: A randomized trial', *Nutrients*, 9: pii: E30.

Radzinsky, V.E., Klimova, O.I., Mingaleva, N.V.*, et al.* (2021) *Combating deficiency: following the new WHO recommendations (2020). Overcoming vitamin and mineral deficiencies as a component of pregnancy management* (StatusPraesens).

Schaefer, E., Bieri, G., Sancak, O., Barella, L. & Maggini, S. (2016) 'A randomized, placebo-controlled trial in women of childbearing age to assess the effect of folic acid and methyl-tetrahydrofolate on erythrocyte folate levels', *Vitam Miner*, 5: 134.

Schaefer, E., Demmelmair, H., Horak, J.*, et al.* (2020) 'Multiple Micronutrients, Lutein, and Docosahexaenoic Acid Supplementation during Lactation: A Randomized Controlled Trial', *Nutrients*, 12.

Sun, N.-X., Xu, C., Zhang, Q., Lu, X.-M. & Li, W. (2013a) 'Impact of multivitamin supplementation on trace element levels in serum and follicular fluid of women undergoing in vitro fertilisation', *J Development Med*, 1: 74-77.

Sun, Y., Dong, X. & Zhang, H. (2013b) 'Clinical analysis on preventive effect of multivitamin on fetal neural tube defect after IVF-ET', *Matern Child Health Care China*, 28: 1199-1203.

Vanderlelie, J. , Scott, R. , Shibl, R. *, et al.* (2016) 'First trimester multivitamin/mineral use is associated with reduced risk of pre-eclampsia among overweight and obese women', *Matern Child Nutr*, 12: 339-348.

Wang, Y., Yu, Q., Chen, R.*, et al.* (2017) 'A comparative study on the administration of multi-vitamin tablets or single folic acid tablets to achieve erythrocyte folic acid threshold concentration', *J Reprod Med*, 26.
